# Genetic and Chemical Screenings Identify HDAC3 as a Key Regulator in Hepatic Differentiation of Human Pluripotent Stem Cells

**DOI:** 10.1016/j.stemcr.2018.05.001

**Published:** 2018-05-31

**Authors:** Shuang Li, Mushan Li, Xiaojian Liu, Yuanyuan Yang, Yuda Wei, Yanhao Chen, Yan Qiu, Tingting Zhou, Zhuanghui Feng, Danjun Ma, Jing Fang, Hao Ying, Hui Wang, Kiran Musunuru, Zhen Shao, Yongxu Zhao, Qiurong Ding

**Affiliations:** 1 CAS Key Laboratory of Nutrition, Metabolism and Food Safety, Shanghai Institute of Nutrition and Health, Shanghai Institutes for Biological Sciences, University of Chinese Academy of Sciences, Chinese Academy of Sciences, Shanghai 200031, P. R. China; 2 CAS Key Laboratory of Computational Biology, CAS-MPG Partner Institute for Computational Biology, Shanghai Institute of Nutrition and Health, Shanghai Institutes for Biological Sciences, Chinese Academy of Sciences, Shanghai 200031, P. R. China; 3 College of Mechanical Engineering, Dongguan University of Technology, Dongguan, Guangdong 523808, P. R. China; 4 School of Public Health, Shanghai Jiao Tong University School of Medicine, Shanghai 200025, P. R. China; 5 Cardiovascular Institute, Department of Medicine, Department of Genetics, Perelman School of Medicine at the University of Pennsylvania, Philadelphia, PA 19104, USA

**Keywords:** human pluripotent stem cells, hepatic differentiation, genome-wide CRISPR/Cas9 screening, histone deacetylase 3, histone deacetylase inhibitor CI-994

## Abstract

Hepatocyte-like cells (HLCs) derived from human pluripotent stem cells (hPSCs) offer a promising cell resource for disease modeling and transplantation. However, differentiated HLCs exhibit an immature phenotype and comprise a heterogeneous population. Thus, a better understanding of HLC differentiation will improve the likelihood of future application. Here, by taking advantage of CRISPR-Cas9-based genome-wide screening technology and a high-throughput hPSC screening platform with a reporter readout, we identified several potential genetic regulators of HLC differentiation. By using a chemical screening approach within our platform, we also identified compounds that can further promote HLC differentiation and preserve the characteristics of *in vitro* cultured primary hepatocytes. Remarkably, both screenings identified histone deacetylase 3 (HDAC3) as a key regulator in hepatic differentiation. Mechanistically, HDAC3 formed a complex with liver transcriptional factors, e.g., HNF4, and co-regulated the transcriptional program during hepatic differentiation. This study highlights a broadly useful approach for studying and optimizing hPSC differentiation.

## Highlights

• Genome-wide genetic screening to identify regulators in hepatic differentiation
• HDAC3 regulates hepatic differentiation
• Small molecule CI-994 treatment improves hepatic differentiation
• A broadly useful approach for studying and the optimization of hPSC differentiation

## Graphical Abstract

**Figure:**
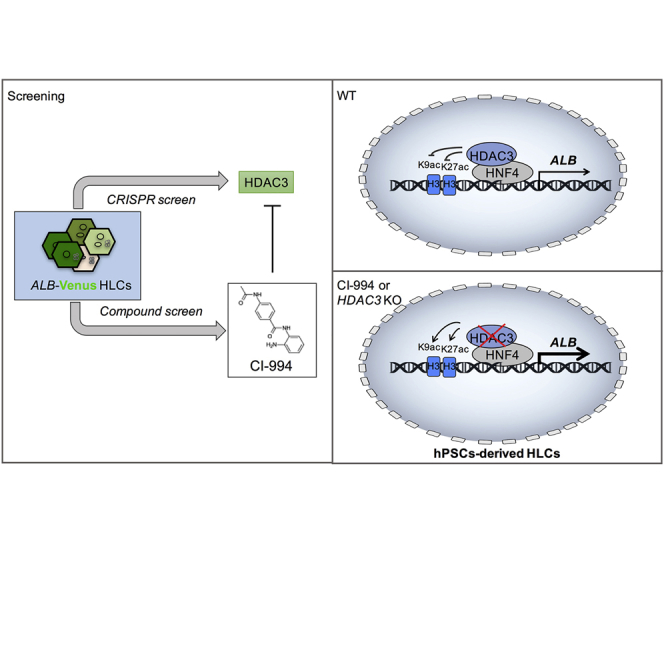

## Teaser

Ding and colleagues developed a broadly useful approach to study novel regulators involved in hepatic differentiation of human pluripotent stem cells, with a combination of reporter system construction, genome-wide CRISPR-Cas9-based genetic screening, and targeted chemical screening. Studies revealed HDAC3 as a key regulator in hepatic lineage determination.

## Introduction

Human pluripotent stem cells (hPSCs) hold great promise as an attractive resource of human somatic cells, due to their ability to self-renew and, theoretically, to differentiate into any of the myriad of somatic cell types in the human body. In practice, the ability to differentiate into a desired cell type often depends on the availability of an efficient protocol. Stepwise protocols using defined factors have been established years ago for the differentiation from hPSCs toward human hepatocytes (Si-Tayeb et al., 2010, Song et al., 2009, Sullivan et al., 2010). However, the resulting hepatocytes exhibit an immature hepatic phenotype (e.g., express fetal markers such as alpha fetoprotein) and remain a heterogeneous population. In addition, substantial variation in differentiation efficiencies has been observed among different hPSC lines. Considerable effort has later been devoted to studying the directed differentiation process or screening for compounds for enhanced maturation of HLCs (Cheng et al., 2012, Li et al., 2017, Loh et al., 2014, Shan et al., 2013). While many of the improvements to the hepatic differentiation protocol are based on the knowledge acquired in understanding signal pathways that control embryonic lineage bifurcations, none of the existing studies tackled the differentiation problems through an unbiased genetic screening approach. With recent advances in genome editing technologies, especially the clustered regularly interspaced short palindromic repeats (CRISPR)/CRISPR-associated (Cas) system, genome-wide genetic screening becomes a high-throughput, low-cost platform that enables comprehensive study of regulators in a biological process (Shalem et al., 2015). In the present study, we devised a reporter system and applied a CRISPR/Cas9-based genetic screening approach to identify potential regulators in hepatic lineage determination. Based on the results from the genetic screening, we also performed a targeted, small-scale chemical screening. We have identified several regulators in hepatic differentiation and an efficient small molecule that can improve the differentiation efficiency. Our study also demonstrated a broadly useful approach for studying hPSC differentiation.

## Results

### Generation of the *ALB-Venus* Reporter Line

**Figure 1:**
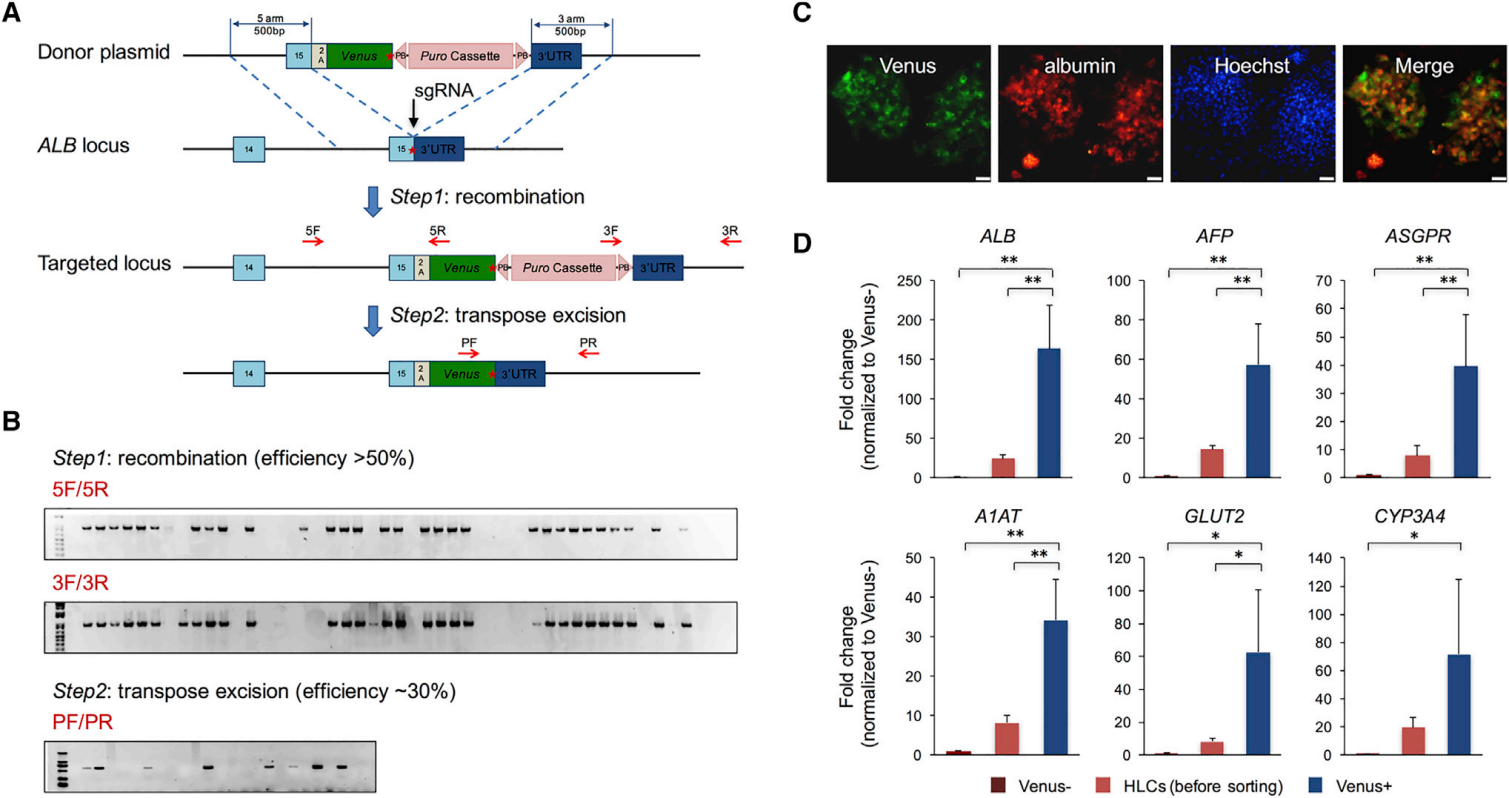
Generation of the *ALB-Venus* Reporter Line (A) Schematic view of the targeting strategy. The red star indicates the stop codon of the *ALB* gene. Arrows indicate screening primers used for genotyping of individual clones. (B) Representative PCR screening results in both recombination and transposon excision steps. (C) Albumin staining of HLCs derived from one positive hPSC clone (scale bar, 50 μm). (D) Gene expression analysis of different cell populations from before or after FACS of HLCs derived from one positive hPSC clone. n = 3 independent experiments. Data are represented as means with SEM. ^∗^p < 0.05, ^∗∗^p < 0.01.

Albumin is regarded as a molecular marker of hepatocytes that can reflect cell maturation. To perform screenings for regulators in HLC differentiation, we developed a reporter hPSC line with a T2A-Venus cassette knocked in before the stop codon of the endogenous *ALB* gene, termed the *ALB-Venus* reporter line (Figure 1A). A previously established induced pluripotent stem cell line, DiPS 1016 SevA (called 1016 for short), with relatively poor HLC differentiation efficiency, was chosen as the parental hPSC line. We designed and screened for an efficient single guide RNA (sgRNA) targeting the site of the *ALB* stop codon. CRISPR-Cas9 homology-directed repair with a donor template, followed by transposon excision yielded cells with one or two knocked-in alleles at a relatively high efficiency (more than 50% at step 1 and ∼30% at step 2) (Figures 1A and 1B). To validate the obtained reporter cell line, we differentiated hPSCs into HLCs using an adapted protocol (Ding et al., 2013, Si-Tayeb et al., 2010). Image analysis of HLCs displayed a very high degree of overlap between the Venus and albumin signals (Figure 1C). HLCs were also sorted based on the intensity of Venus expression to compare three cell populations: Venus−, Venus+, and presorted HLCs; and Venus+ cells had significantly higher expression levels of several hepatic markers compared with either presorted HLCs or Venus− HLCs (Figure 1D).

### Genetic Screenings Reveal that HDAC3 Is Involved in the Regulation of HLC Differentiation

**Figure 2:**
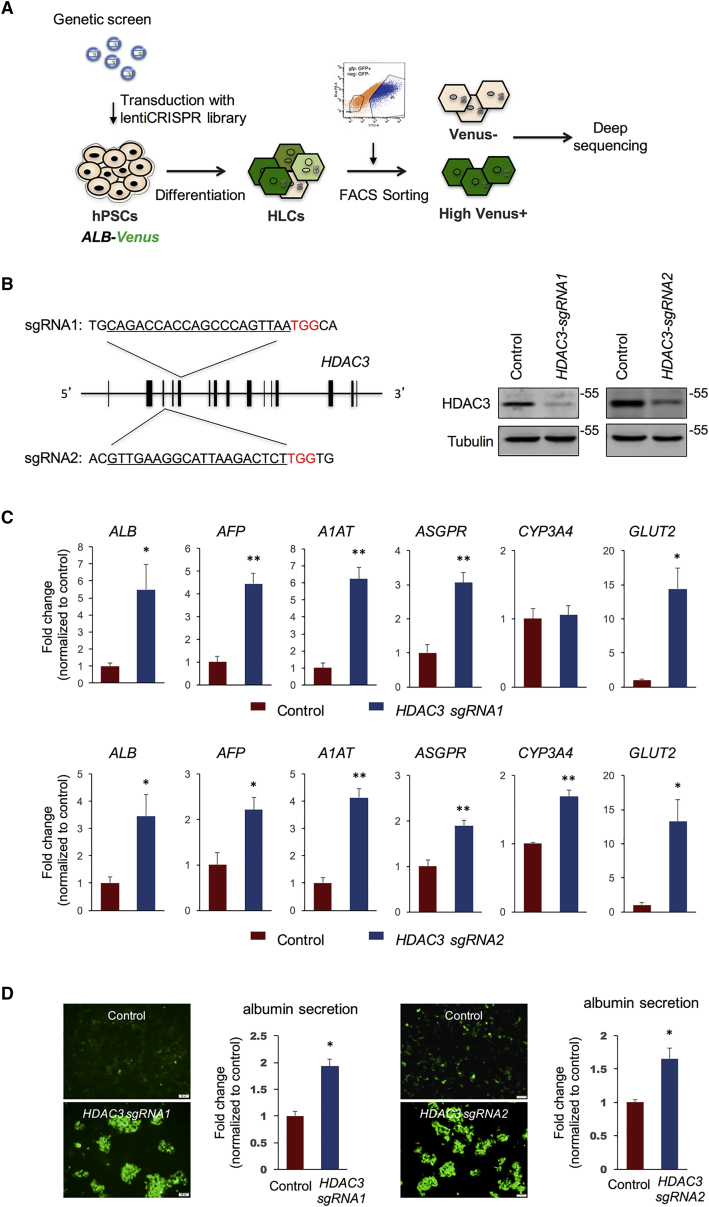
Genetic Screenings Reveal that *HDAC3* Is Involved in the Regulation of HLC Differentiation (A) Schematic view of the genetic screening strategy. See also Figures S1A and S1B. (B) SgRNA sequences targeting human *HDAC3* gene (underlined part, left); western blot analysis of HDAC3 protein in CRISPR-Cas9-treated HLCs and control cells. (C) Gene expression analysis of CRISPR-Cas9-treated HLCs and control cells from three independent experiments. (D) Representative images of Venus intensity in HLCs treated with CRISPR-*HDAC3* and control viruses (scale bar, 50 μm); albumin mass measured by ELISA in media collected from HLCs treated with CRISPR-*HDAC3* or control viruses, normalized to mean levels of control group (n = 3 independent experiments). Data are represented as means with SEM. ^∗^p < 0.05, ^∗∗^p < 0.01. See also Figures S1C and S1D.

Using the *ALB-Venus* reporter cell line, we set up a genome-wide genetic screening by using the GeCKO library (Shalem et al., 2014) (Figure 2A). hPSCs were transduced by the GeCKO library and differentiated toward HLCs. HLCs were then sorted to obtain high Venus+ (top 5%) and Venus– (bottom 5%) populations. Two other cell groups, presorted HLCs and hPSCs, were also collected. Each population of cells was subjected to genomic DNA isolation and deep sequencing of integrated sgRNAs. Candidate genes were initially screened for significant enrichment of sgRNAs in the Venus+ population compared with the presorted HLCs population. The sgRNA counts in the other two control groups, the Venus– HLCs and hPSCs, were also referenced when winnowing the list of candidate genes. Five genes stood out as top candidates: *TIMP3*, *RAB3GAP1*, *ATG7*, *RPS6KA2*, and *HDAC3* (Figures S1A and S1B). To validate these candidate genes, individual gene-knockout hPSCs were generated by CRISPR-Cas9-mediated targeting and subjected to HLC differentiation. Among the five tested candidate genes, *ATG7*^−/−^, *RPS6KA2*^−/−^, and *HDAC3*^−/−^ hPSCs displayed significantly increased differentiation efficiency compared with control cells (Figures 2B–2D, S1C, and S1D). On the other hand, we did not observe obvious changes in HLCs from *TIMP3*^−/−^ and *RAB3GAP1*^−/−^ hPSCs (data not shown).

### Chemical Screenings Identify that CI-994 Improves HLC Differentiation

Of the three genes we successfully validated, *ATG7* encodes autophagy-related protein 7 (Ohsumi, 2001); *RPS6KA2* encodes ribosomal protein S6 kinase alpha-2, a member of the ribosomal S6 kinase family of serine and threonine kinases that signals downstream of the mitogen-activated protein kinase pathway (Bignone et al., 2007); and *HDAC3* encodes histone deacetylase 3, a class I histone deacetylase that is a core component of nuclear receptor corepressor complexes and was previously found to regulate liver triglyceride homeostasis (Papazyan et al., 2016, Sun et al., 2012) and be critical for maintaining genome stability in liver cells (Bhaskara et al., 2010). With the goal of directly translating these findings into improvements of HLC differentiation protocol, we next assessed whether small molecules can reproduce the effects caused by reduced expression of these genes. We first tested two autophagy inhibitors (wortmannin and chloroquine), as well as an inhibitor of ribosomal protein S6 kinase (BID1870) (Sapkota et al., 2007). We noticed slightly improved differentiation of HLCs by wortmannin treatment (Figure S2A), whereas no significant improvement by chloroquine or BID1870 treatment (data not shown).

**Figure 3:**
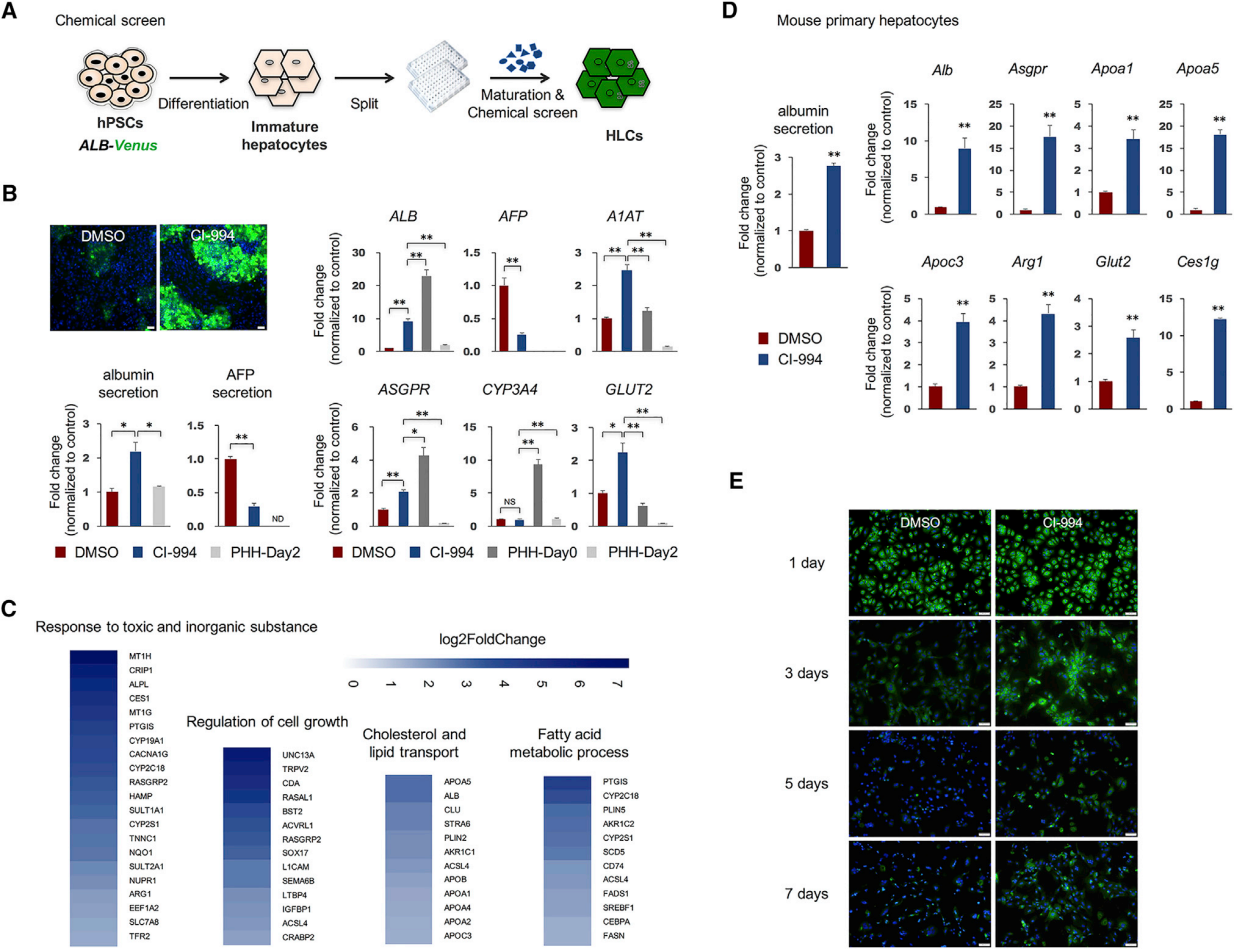
Chemical Screenings Identify that CI-994 Improves HLC Differentiation and Delays the Loss of Hepatocyte Characteristics of Primary Hepatocytes Cultured *In Vitro* (A) Schematic view of the chemical screening strategy. (B) Representative images of Venus intensity in HLCs treated with 10 μM CI-994 or DMSO control (scale bar, 50 μm) (above left); albumin and AFP mass measured by ELISA in media collected from HLCs treated with 10 μM CI-994 or DMSO control and PHH-day2, normalized to mean levels of control group (below left); gene expression analysis of 10 μM CI-994-treated HLCs, control HLCs, PHH-day0 and PHH-day2 (right). n = 3 independent experiments. ND, not determined; NS, not significant. See also Figures S2, S3A, and S3B. (C) RNA-seq analysis of 10 μM CI-994-treated HLCs and control cells. Shown are genes upregulated in CI-994-treated cells compared with control cells. (D) Albumin mass measured by ELISA in media collected from primary mouse hepatocytes cultured *in vitro* and treated with 10 μM CI-994 or DMSO control for 7 days, normalized to mean levels of control group (left); gene expression analysis of above cell groups (right). n = 3 independent experiments. (E) Representative albumin staining of primary hepatocytes treated with 10 μM CI-994 or DMSO control for indicated days (scale bar, 50 μm). Data are represented as means with SEM. ^∗^p < 0.05, ^∗∗^p < 0.01. See also Figure S3C.

The involvement of *HDAC3* in HLC differentiation suggested that inhibition of histone deacetylation can improve HLC differentiation. Rather than testing individual compounds, we next devised a chemical screening platform with the *ALB-Venus* reporter line. As HLCs are typically stacked in multiple layers in a culture dish at the end of more than 20 days of differentiation, complicating the ability to perform image-based screening, we split the cells at the stage of immature hepatocytes and re-plated in a single layer in 96-well plates for further maturation and chemical screening (Figure 3A). In total, 43 epigenetic regulators were screened; treatment with CI-994, which represents a selective inhibitor of class I HDACs (Undevia et al., 2004), resulted in a dramatic increase in Venus expression intensity as well as percentage of positive cells; whereas an inhibitor to histone acetyltransferases (HAT), Garcinol, resulted in the lowest Venus expression (Figure S2B).

Further analysis showed that HLCs treated with CI-994 displayed significantly higher expression levels of several hepatic markers, as well as reduced expression of the fetal liver marker *AFP*, compared with control cells (Figures 3B, S3A, and S3B). Primary human hepatocytes (PHHs) were also included for comparison. As primary hepatocytes lose hepatic characteristics rapidly when cultured *in vitro*, both freshly isolated cells (PHH-Day0) and cells after culturing *in vitro* for 2 days (PHH-Day2) were included. Results indicated that after CI-994 treatment, HLCs remained immature when compared with PHH-day0; however, better performed PHH-day2 in some aspects, such as increased albumin secretion (Figure 3B). In the meantime, CI-994 treatment outperformed *HDAC3*^−/−^ hPSCs, as reflected by reduced AFP expression, we thus suspect that HDAC1 and 2 may also involve in hepatic differentiation, albeit they were not picked up by the genetic screening. Possible reasons may lie in that the screening was initially designed to test effects of individual genes, and there may exist compensation effects between HDAC1 and 2. RNA sequencing (RNA-seq) analysis was next performed on HLCs. CI-994 treatment led to 182 upregulated genes and 34 downregulated genes with a cutoff of false discovery rate (FDR) ≤0.01 and fold change ≥2. Several GO terms relevant to hepatic functions were significantly enriched in upregulated genes (Figure 3C). No GO term showed significant enrichment in downregulated genes.

### CI-994 Treatment Also Delays the Loss of Hepatocyte Characteristics of Primary Hepatocytes Cultured *In Vitro*

Besides the differentiation of HLCs from hPSCs, a complementary approach to studying hepatocyte function *in vitro* is to isolate and culture primary hepatocytes. However, primary hepatocytes are known to rapidly de-differentiate and lose hepatic characteristics *in vitro*, although several culture conditions that can maintain hepatocytes in culture for a limited time period have been reported (Block et al., 1996, Mitaka et al., 1991, Mizuguchi et al., 2001, Richman et al., 1976). We next asked whether CI-994 can also delay the de-differentiation of primary hepatocytes *in vitro*. Indeed, we found that primary hepatocytes treated with CI-994 better maintained their morphology and hepatic gene expression compared with control cells (Figures 3D, 3E, and S3C). These results suggest that CI-994 might be useful for the maintenance of primary hepatocytes in culture.

### HDAC3 Coordinates with HNF4 in Regulating *ALB* Expression in HLCs

**Figure 4:**
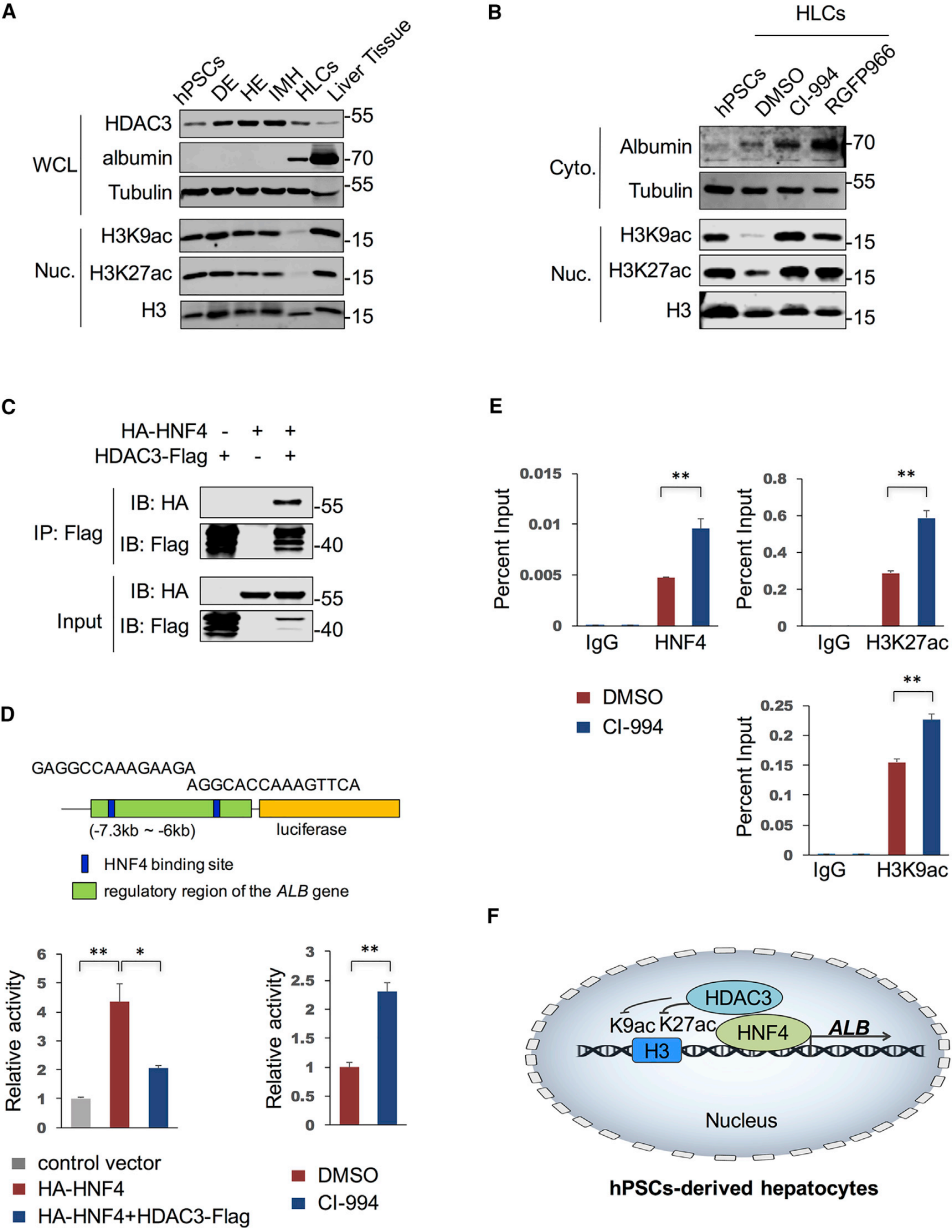
HDAC3 Coordinates with HNF4 in Regulating *ALB* Expression in HLCs (A) Western blot analysis of cells at different stages in hepatic differentiation and primary human liver tissue. WCL, whole cell lysates; DE, definitive endoderm; HE, hepatic endoderm; IMH, immature hepatocytes. (B) Western blot analysis of hPSCs and HLCs treated with CI-994 (5 μM) or RGFP966 (10 μM) or DMSO as control. The cytosol (Cyto.) and nuclear (Nuc.) sections of each sample in (A) and (B) were separated for analysis. (C) Co-immunoprecipitation analysis of HNF4 and HDAC3 in HEK293T cells. (D) Luciferase reporter assay in HEK293T cells (n = 3 independent experiments). The schematic cartoon of the luciferase reporter construction was shown above. (E) ChIP analysis with indicated antibodies in HLCs treated with DMSO or CI-994 (5 μM) from three independent experiments. Primers used in qPCR analysis amplified the *ALB* enhancer region (−7 to −6.5 kb). (F) The schematic diagram of a working mechanism that HDAC3 coordinates with HNF4 in regulating *ALB* expression in HLCs. Data are represented as means with SEM. ^∗^p < 0.05, ^∗∗^p < 0.01.

Both the genetic and chemical screenings suggest that inhibition of class I HDACs, specifically HDAC3, improves hepatic differentiation of hPSCs. We noticed a dynamic change of HDAC3 expression levels along the hepatic differentiation process and a dramatic decrease of both H3K9ac and H3K27ac nuclear signals at HLC stage, in comparison with signals in cells from other stages as well as in the primary human liver tissue (Figure 4A). Treatment with CI-994 or RGFP966—a specific inhibitor to HDAC3—reversed the levels of both H3K9ac and H3K27ac, and enhanced the expression of albumin in HLC stage (Figure 4B). It is well known that acetylation of H3K9 and H3K27 mark actively transcribed regions in genome (Lawrence et al., 2016), which can be deacetylated by HDACs, resulting in reduced local gene transcription (Allis and Jenuwein, 2016). These results suggest that the low histone acetylation level in HLC stage as part of the causation of compromised hepatic gene expression in HLCs compared to human primary liver tissue.

Hepatocyte differentiation is dependent on activation of a series of hepatic transcription factors, such as HNF4 and CEBP (Li et al., 2000, Nagy et al., 1994). Since HNF4 was found prominently at co-occupied sites with HDAC3 on the mouse liver genome (Armour et al., 2017, Papazyan et al., 2016), we next investigated whether HDAC3 regulated hepatic gene expression, e.g., albumin, through interacting with HNF4. Indeed, our results confirmed the interaction between HNF4 and HDAC3 (Figure 4C). We further assessed whether HDAC3 and HNF4 co-regulate the albumin expression. Several HNF4 binding sites were predicted in the enhancer region of the *ALB* gene (−7.3 kb to −6 kb from transcription start site [TSS]). We cloned this regulatory region into a luciferase reporter plasmid. We found that HNF4 overexpression significantly enhanced the luciferase signal, which could be attenuated by HDAC3 overexpression. And CI-994 treatment increased the luciferase signal (Figure 4D). Chromatin immunoprecipitation (ChIP)-qPCR analysis further confirmed that CI-994 treatment also increased HNF4 binding activity and both signals of H3K9ac and H3K27ac in the *ALB* enhancer region in HLCs (Figure 4E). Altogether, these results indicated a working mechanism that the epigenetic regulator HDAC3 coordinates with hepatic transcription factors, e.g., HNF4, in regulating the expression of lineage-specific genes during hepatic differentiation of hPSCs (Figure 4F). It is interesting to read in a recent publication that HDAC3 organizes heterochromatin at the nuclear lamina and therefore regulates the cardiac differentiation of mouse embryonic stem cells (mESCs) (Poleshko et al., 2017). A deeper understanding to the role of HDAC3 in hepatic differentiation warrants further investigation.

## Discussion

It remains a general problem in the stem cell field that somatic cells derived from hPSCs are all at a relatively immature stage, and many *in vitro* differentiation systems require further optimization. We addressed this problem by developing a cellular platform that could be applied to high-throughput screening for both genetic factors and chemical compounds that improve differentiation. This approach can readily be adapted to improve other differentiation protocols. We also note several limitations of the approach. (1) Reporter cell lines only reflect a limited part of the functionality of cells. For example, the *ALB-Venus* reporter line used in our study reflects only the expression of albumin protein, which does not necessarily represent fully mature hepatocytes. Indeed, in our study, HLCs with either genetic ablation of HDAC3 or CI-994 treatment showed significantly increased *ALB* expression; however, remained at a relatively immature stage with high *AFP* expression and low CYP3A4 expression compared with PHHs. Dual- or multiple-reporter cell lines (e.g., *CYP3A4* as a positive reporter, *AFP* as a negative reporter) could potentially be used to better identify drivers of cell maturity. (2) As most terminal differentiated cells lack the ability to proliferate, the selection step (e.g., the fluorescence activating cell sorting [FACS] step in our study) is less able to enrich for the desired sgRNAs compared with studies in which enhancement of proliferation or acquisition of drug resistance is the desired outcome (Shalem et al., 2014). Because of this, studies with a larger number of cells (or fewer sgRNAs in the library) are needed to identify true positive enriched sgRNAs and eliminate false-negative sgRNAs. (3) Lengthy, multi-step differentiation protocols make it more difficult to screen for factors that will improve cell maturity. For example, in our study, cells were infected with CRISPR lentivirus at the undifferentiated stage and analyzed at the terminal HLC stage, making it harder to identify factors that would have the desired effect when modulated only in a specific stage of differentiation. An inducible CRISPR-Cas9-based platform could potentially define contributors in specific differentiation stages by modulating the time course of gene knockdown (Chen et al., 2015, Gonzalez et al., 2014). These limitations notwithstanding, by combining an hPSC reporter cell line, a CRISPR-Cas9-mediated genetic screening, and a chemical screening, our study highlights a broadly useful approach for the optimization of hPSC differentiation.

## Experimental Procedures

### Construction of the *ALB-Venus* Reporter Line

Construction of the *ALB-Venus* reporter line was following a similar protocol as described (Ding et al., 2013). Details are given in Supplemental Experimental Procedures.

### Genome-wide CRISPR-Cas9 Screenings

In general, the *ALB-Venus* reporter hPSCs were transduced by the CRISPR lentivirus library, followed by hepatic differentiation to obtain HLCs. Cells with high Venus expression (top 5%) were then collected by FACS sorting, and different control groups, including Venus− cells, HLCs, and hPSCs, were also obtained. Genomic DNA from different cell groups was later extracted and subjected to PCR amplification and deep sequencing. sgRNAs and corresponding genes significantly over- or under-represented in the samples of interest were identified. Details are given in Supplemental Experimental Procedures.

### Chemical Screenings

The *ALB-Venus* reporter hPSCs were differentiated to get immature hepatocytes. Cells were then split and plated in 96-well plates for further maturation and chemical screening. Cells were then analyzed for Venus expression in a high-throughput platform using High Content Screening (Cellomics ArrayScan VTI; Thermo Fisher Scientific). Details are given in Supplemental Experimental Procedures.

### Cell Culture

Cell culture was following a standard protocol. Details are given in Supplemental Experimental Procedures.

### Lentivirus Packaging and Generation of CRISPR-Cas9 Knockout Cell Lines

Lentivirus packaging and generation of CRISPR-Cas9 knockout cell lines were following standard methods. Details are given in Supplemental Experimental Procedures.

### Differentiation of hPSCs into HLCs

Differentiation was performed following the protocols of Si-Tayeb et al. (2010). Details are given in Supplemental Experimental Procedures.

### ELISAs, Immunocytochemistry, and Western Blot Analysis

These procedures were performed using standard methods. Details are given in Supplemental Experimental Procedures.

### Quantitative RT-PCR

Quantitative RT-PCR was performed using standard methods. Details and oligonucleotide sequences are given in Supplemental Experimental Procedures.

### Plasmid Construction, Co-immunoprecipitation, Dual-Luciferase Reporter Assay, and ChIP

These procedures were performed using standard methods. Details are given in Supplemental Experimental Procedures.

### RNA-Seq and Statistical Analysis

RNA-seq and statistical analysis were performed using standard methods. Details are given in Supplemental Experimental Procedures.

## Author Contributions

S.L., Y.Z., and Q.D. designed the research. S.L. performed all experiments. M.L. performed data analysis for CRISPR screening and RNA-seq experiments. X.L., Y.Y., Y.W., Y.C., Y.Q., T.Z., Y.Z., Z.F., D.M., J.F., H.Y., H.W., and K.M. assisted with either experiments or data analysis. S.L., M.L., H.Y., K.M., Z.S., Y.Z., and Q.D. wrote the manuscript. Z.S., Y.Z., and Q.D. supervised the project.

## Accession Numbers

Raw data were deposited in the NCBI Sequence Read Archive with the accession number SRA: SRP139546.

## Supplemental Information

Document S1. Supplemental Experimental Procedures and Figures S1–S3

Document S2. Article plus Supplemental Information
