# Supplementary material for: Genetic and Chemical Screenings Identify HDAC3 as a Key Regulator in Hepatic Differentiation of Human Pluripotent Stem Cells
Source: Stem Cell Reports. 2018 May 31;11(1):22–31. doi: 10.1016/j.stemcr.2018.05.001 (PMC6066908; doi:10.1016/j.stemcr.2018.05.001)
Supplement: Document S1. Supplemental Experimental Procedures and Figures S1–S3 [file mmc1.pdf]

Stem Cell Reports, Volume *11*

Supplemental Information

Genetic and Chemical Screenings Identify HDAC3 as a Key Regulator in Hepatic Differentiation of Human Pluripotent Stem Cells

Shuang Li, Mushan Li, Xiaojian Liu, Yuanyuan Yang, Yuda Wei, Yanhao Chen, Yan Qiu, Tingting Zhou, Zhuanghui Feng, Danjun Ma, Jing Fang, Hao Ying, Hui Wang, Kiran Musunuru, Zhen Shao, Yongxu Zhao, and Qiurong Ding

Supplemental Information

Inventory of Supplemental Information

Supplemental Figures S1-S3 and Legends

Figure S1 related to main Figure 2

Figure S2-3 related to main Figure 3

Supplemental Experimental Procedures

Supplemental References

Supplemental Figures

Figure S1. Related to main Figure 2.

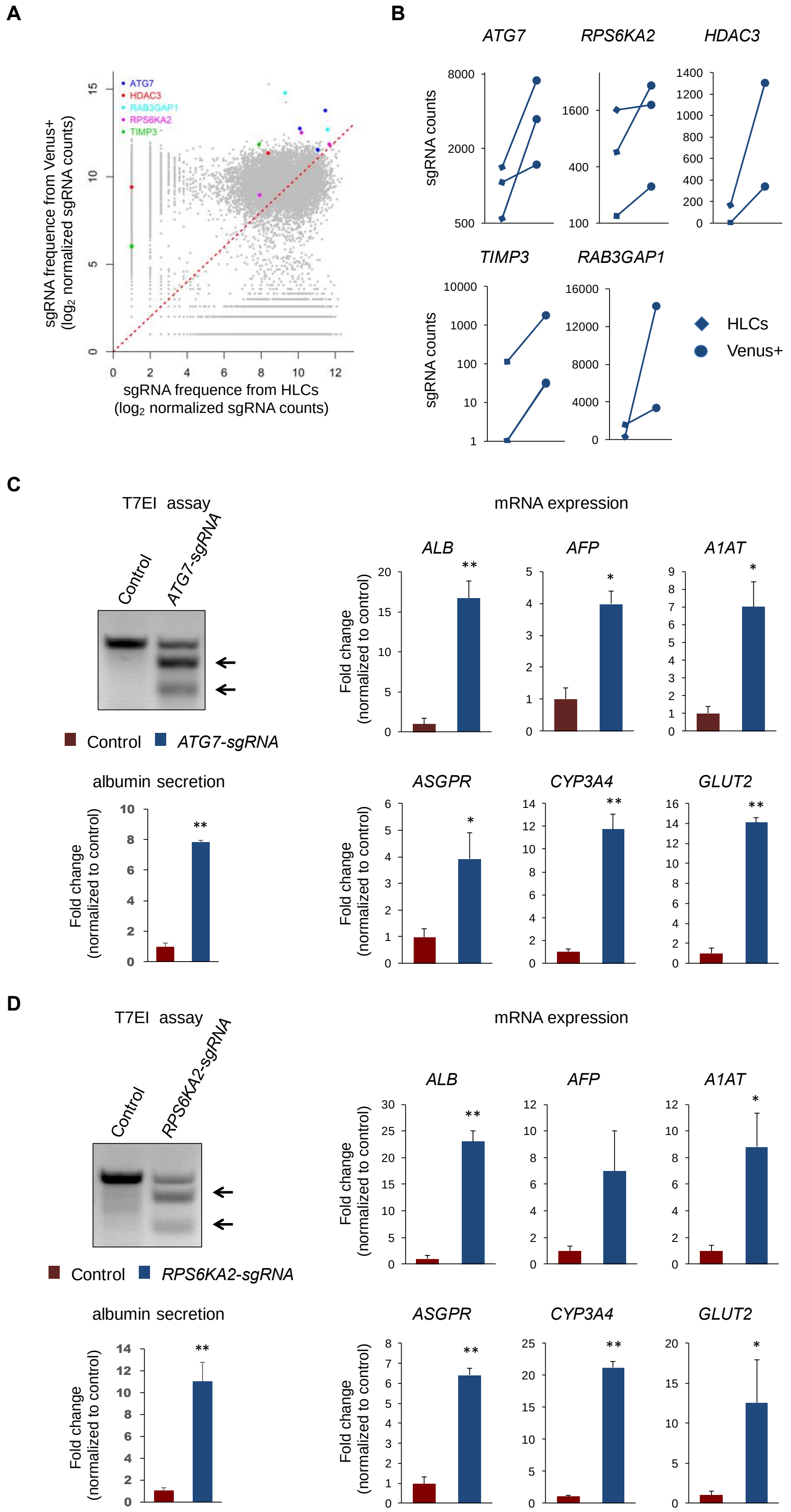

Figure S2. Related to main Figure 3.

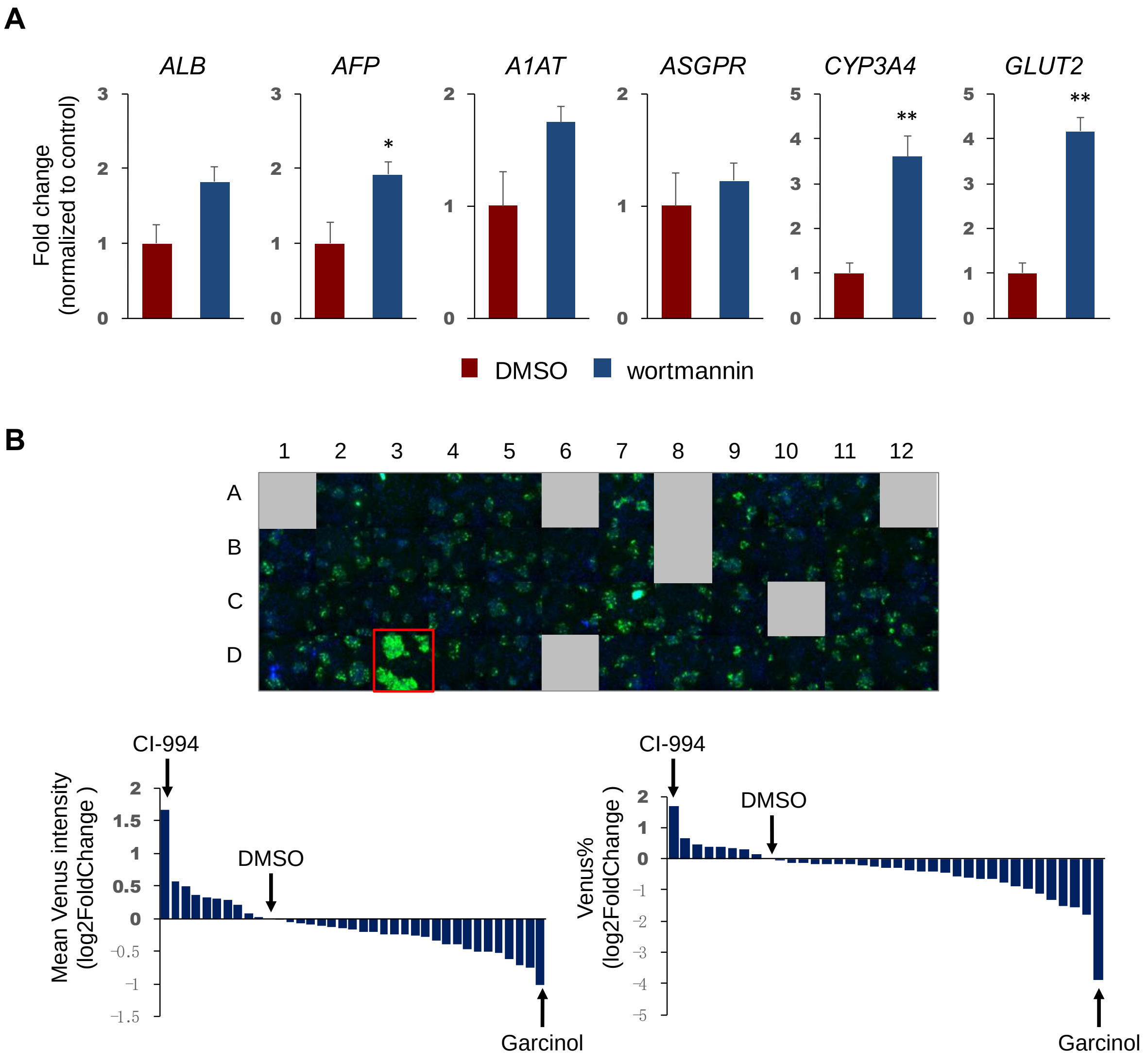

Figure S3. Related to main Figure 3.

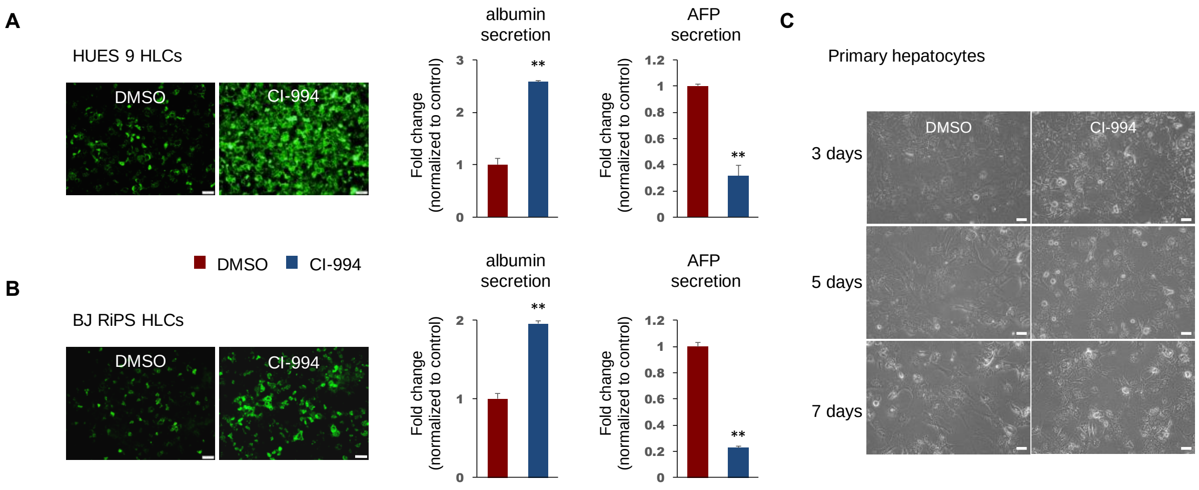

Supplemental Figure Legends

Figure S1. Genetic screenings identify several genes that are involved in regulation of HLC differentiation. Related to main Figure 2. (**A**) Scatterplot showing enrichment of specific sgRNAs in Venus+ group compared to HLC group. (**B**) sgRNA counts of indicated genes in HLC and Venus+ groups from the GeCKO screening. Counts of the third sgRNA each of *HDAC3* and *RAB3GAP1* was zero so was omitted in the graph. Two of the three sgRNAs of *TIMP3* showed similar counts and was overlapped in the graph. (**C**) T7EI analysis of HLCs treated with CRISPR-*ATG7* or control viruses. Arrows show the cleavage products resulting from the T7EI assays (above left); albumin mass measured by ELISA in media collected from HLCs treated with CRISPR-*ATG7* and control viruses, normalized to mean levels of control group (n = 3 independent experiments) (below left); gene expression analysis of CRISPR-Cas9-treated HLCs and control cells (n = 3 independent experiments) (right). (**D**) Same set of experiments as listed in (**C**) performed with CRISPR-*RPS6KA2* (n = 3 independent experiments). Data are represented as means with SEM. * P<0.05, ** P<0.01.

Figure S2. Chemical screenings identify that CI-994 improves HLC differentiation. Related to main Figure 3. (**A**) Gene expression analysis of 50 nM wortmannin-treated HLCs and control cells (n = 3 independent experiments). (**B**) Representative image of a half 96-well plate with Venus signal (green) and nuclei staining (blue) (above); mean Venus intensity (left) and percentage of Venus positive cells (right) as normalized to values in control wells treated with DMSO (below). Cells in D8-D12 wells were treated with DMSO as controls. There was no signal from some wells such as A1, A6, *etc*. due to cell death after chemical treatment. The well with CI-994 treatment was marked with red box.

Figure S3. Effects of CI-994 treatment in HLC differentiation and maintenance of primary hepatocytes in culture. Related to main Figure 3. (**A**) Representative albumin staining of HLCs derived from HUES 9 treated with 5 μM CI-994 or DMSO control (left); albumin and AFP mass measured by ELISA in media collected from HLCs treated with 10 μM CI-994 or DMSO control, normalized to mean levels of control group (right). N = 3 independent experiments. (**B**) Representative albumin staining of HLCs derived from BJ RiPS treated with 10 μM CI-994 or DMSO control (left); Albumin and AFP mass measured by ELISA in media collected from HLCs treated with 10 μM CI-994 or DMSO control, normalized to mean levels of control group (right). N = 3 independent experiments. (**C**) Representative images of primary hepatocytes in bright field treated with 10 μM CI-994 or DMSO control for indicated days (scale bar = 50 μm). Data are represented as means with SEM. * P<0.05, ** P<0.01.

Supplemental Experimental Procedures

Cell culture

Human embryonic kidney 293T cells were maintained in DMEM supplemented with 10% fetal bovine serum and 1% penicillin / streptomycin. 1016 iPSC, BJ RiPS, and HUES 9 (HSCI iPS Core, Harvard)(Cowan et al., 2004) were grown in feeder-free adherent culture in chemically defined mTeSR1 (STEMCELL Technologies, 05850) supplemented with penicillin and streptomycin. Plates were precoated with Geltrex matrix (Invitrogen, A1413202). The cells were disassociated with accutase (Invitrogen, A1110501) and regularly passaged. Mouse primary hepatocytes were isolated from 8 to 10 weeks old mice following a procedure as decribed(Dentin et al., 2004). Cells were then seed on plates precoated with collagen (Sigma, C3867-1VL) at a density of 2×10^5 per well in 12-well plates, and maintained in hepatocyte medium (Gibco, 17705-021) supplemented with 2 mM L-Glutamine (Gibco, 25030) and 2% penicillin / streptomycin. Primary human hepatocytes were purchased from BioreclamationIVT (BioIVT, M00995-P).

Lentivirus packaging

To prepare lentiviruses or the CRISPR lentivirus library, HEK293T cells in each 15-cm dish were transfected with 22.5 μg CRISPR plasmids together with 14.7 μg pMDL, 5.7 μg pRev and 7.9 μg pVSVG packaging plasmids. After transfection, medium with viral particles were collected 48 hours and 72 hours later and centrifuged at 20,000 r.p.m at 4 ℃ for 2 hours to pellet viral particles. Viral pellets were then re-suspended in DMEM at 4 ℃ overnight and titer was calculated using a PCR based titration kit (Applied Biological Materials Inc, LV900).

Construction of the *ALB-Venus* reporter line

For construction of the targeting plasmid, 500-bp homology arm directly upstream of the stop codon of human *ALB* gene was synthesized and ligated to the *Venus* cDNA sequence through a T2A sequence, which was used as 5' arm; 500-bp homology arm downstream of the stop codon of human *ALB* gene was synthesized and used as 3' arm. Both arms were subcloned into the PB-MV1Puro-TK vector (Transposagen) by Gibson assembly (NEB, E2611S). sgRNA targeting the human *ALB* gene stop codon locus (5'-AATGTGATGTTATAAGCCTA-3') was synthesized and cloned into lentiCRISPR v2 vector purchased from Addgene (#52961).

Generation of *ALB-Venus-puromycin* recombinant clones was following a similar protocol as described(Ding et al., 2013). Briefly, wild-type 1016 iPSCs were maintained as described above. For targeting, the cells were disassociated into single cells with accutase, and 10 million cells were electroporated with a mix of 30 μg of the CRISPR plasmid and 30 μg of the donor plasmid in a single cuvette (Bio-Rad). The cells were then plated and treated with 1 μg/ml puromycin for 2 days. When single colonies appeared, colonies were manually picked and replated individually to wells of 96-well plates. Colonies were allowed to grow to near confluence over the next 7 days, at which point they were split and replica-plated. Genomic DNA was extracted in 96-well format from one of the plates in lysis buffer (10 mM Tris pH 7.5, 10 mM EDTA, 10 mM NaCl, 0.5% Sarcosyl) containing proteinase K at 56 ℃ overnight in a humidified chamber. Genomic DNA was precipitated by the addition of 95% ethanol containing 75 mM NaCl for 1 hr at room temperature. The DNA was then washed two times in 70% ethanol, allowed to dry at room temperature, and then resuspended in nuclease-free water.

Genotyping to confirm the successful recombination was performed by a PCR method using 2 × Taq mixture (Transgen Biotech, AS111-11). Primer pairs used were as following: for 5' arm inserting: 5'-GGAGGCTTTGTACATGTGGG-3' (outside 5' arm) and 5'-GCTGAACTTGTGGCCGTTTA-3' (inside Venus); for 3' arm inserting: 5'-TCTTCTGTTGGGCTAGGCAA-3' (outside 3' arm) and 5'-CTAAATGCACAGCGACGGAT-3' (inside puromycin). PCR products from positive colonies were further validated by Sanger sequencing. Colonies with successful recombination were recovered from 96-well plates and expanded for puromycin cassette excision in the next step.

Excision-only piggyBac transposase expression was introduced next in positive colonies from recombination step for puromycin cassette excision. Briefly, the cells were disassociated into single cells and 10 million cells were electroporated with 30 μg pPBx-GFP plasmid (Transposagen). Seventy-two hours after electroporation, cells with GFP expression were collected by FACS and replated on 10 cm tissue-culture dishes at around 20,000 cells / dish to allow for recovery in growth media. Single colonies were then manually picked and replated individually to wells of 96-well plates. Genomic DNA and genotype of single colonies were performed as described above. Primers to confirm the successful excision of puromycin cassette were as following: 5'-AAGCTGACCCTGAAGCTCAT-3' (inside Venus) and 5'-TCTTCTGTTGGGCTAGGCAA-3' (outside 3' arm);

5’-ACCGAGCTGCAAGAACTCT-3’ and 5’-TCGTAGAAGGGGAGGTTGC-3’ (both inside puromycin). Colonies with successful puromycin excision were expanded for further experiments.

Genome-wide CRISPR-Cas9 screenings

The lentiviral sgRNA plasmid library for genome-wide CRISPR-Cas9 screening was purchased from Addgene (#1000000048). Library was amplified following the protocol provided by Addgene, and lentiviruses were prepared and titer was calculated as described above.

A total of 4.5×10^7 *ALB-Venus* reporter hPSCs were infected by the lentivirus library at an MOI of 0.7. Cells were treated with puromycin (1 μg/ml) for 2 days to eliminate non-infected cells. Cells were recovered for one more day after puromycin treatment, and subjected to HLC differentiation. Cells were harvested at day 5 at maturation stage and high Venus positive cells (top 5%) were collected by FASC sorting (FACSAria II; BD Biosciences). Control cells, including Venus negative cells (bottom 5%, collected by FACS sorting), HLCs (collected before FACS sorting) and infected hPSCs (collected after puromycin treatment) were obtained at indicated time points.

Genomic DNA of cells from different groups was extracted and the sgRNAs were amplified by PCR method using KOD DNA polymerase (TOYOBO, KOD-401). Briefly, in total 2.2 μg (200 ng per PCR reaction; 11 separate reactions for each sample) of genomic DNA from each group were used as DNA template; the PCR program used was 94 ℃ 5min, 98 ℃ 20s, 58 ℃ 30s, 68 ℃ 12s, 32 cycles. Products (158bp) were gel-purified and quantified. In total 1.4 μg PCR products from each group were pooled together and sent for deep sequencing (Illumina HiSeq4000 system) by using the pair-ended 150bp sequencing protocol. PCR primers used for amplification were: 5’-TGAAAGTATTTCGATTTCTTGGCTT-3’, 5’-CGGTGCCACTTTTTCAAGTT-3’. An 8bp barcode for multiplexing of different biological samples were added at 5’ of each primer.

For data analysis, the sequencing reads of sgRNAs from different samples were first identified by barcode using cutadapt (v1.9) with default parameters. Build-index function of Bowtie(Langmead et al., 2009) was applied on the sgRNA sequences of GeCKO library to generate Burrows-Wheeler index. The sgRNA sequences were then retrieved and counted by aligning processed reads of each sample to the sgRNA library using Bowtie. Maximum 2 mismatches were allowed and only the reads with unique alignment were reported. SgRNAs and corresponding genes significantly over- or under-represented in the samples of interest were identified by MAGeCK(Li et al., 2014) package with the median normalization option compared to the control samples.

Chemical screenings

The *ALB-Venus* reporter hPSCs were differentiated to get immature hepatocytes. Cells were then split with accutase and plated at a density around 5×10^4 cells per well in 96-well plates. After attachment, cells were cultured in maturation medium supplemented with different chemicals for 7 - 9 days. Medium was changed each day. Cells were then analyzed for Venus expression in a high-throughput platform using High Content Screening (HCS) (Cellomics ArrayScan VTI; Thermo Fisher Scientific). Chemical library was purchased from the National Compound Resource Center (Shanghai) and applied in a final concentration of 10 μM of each chemical in screening; Cells treated with DMSO were used as controls.

In experiments testing individual chemicals, immature hepatocytes differentiated from 1016 iPSC, HUES 9 or BJ RiPS as indicated were split and plated at a density around 4×10^5 cells per well in 12-well plates. Cells were then cultured in maturation medium supplemented with individual chemicals or DMSO as controls. Cells were analyzed after 7 - 9 days. Chemicals used in our study were as following: wortmannin (50 nM, Selleck, S2758), chloroquine (20 μM, Selleck, S4157), RPS6KA2 inhibitor (10 μM, Selleck, S2843), CI-994 (5 μM or 10 μM as indicated, Selleck, S2818), and RGFP966(10 μM, Selleck, S7229)

Generation of CRISPR-Cas9 knockout cell lines

The 20bp sequence of sgRNA targeting individual genes was inserted to lentiCRISPR v2 plasmid and used for lentivirus packaging. The target sequences used are 5’-TAGGGTCCATACATTCACTG-3’ for human *ATG7*, 5’-GCAGGAAGAAGGCGTCGTGA-3’ for human *RPS6KA2*, 5’-CAGACCACCAGCCCAGTTAA-3’ for human *HDAC3-sgRNA1* and 5’-GTTGAAGGCATTAAGACTCT-3’ for human *HDAC3-sgRNA2*.

Lentiviruses carrying CRISPR-Cas9 targeting individual genes or empty lentiCRISPR v2 vector as control viruses were packaged. The *ALB-Venus* reporter hPSCs were infected and selected with puromycin (1 μg/ml). Cells were next subjected to genomic DNA extraction for T7EI analysis (NEB, E3321) or to protein extraction for western blot to determine gene editing efficiency. Primers used in T7EI analysis for each gene were as following: 5’-AGGTCGTTGCTTGATCTGCT-3’ and 5’-GTACAGGTACGCTGGTGGTC-3’ for human *ATG7*, 5’-

GGCTCCAACGGCATTTTGTT-3’ and 5’-CTCCACTTTCAAACTCCAGCG-3’ for human *RPS6KA2*, 5’-AATTCCCCTCCCAGCTGC-3’ and 5’-AGGGTTCCAACTTGTTCCTCT-3’ for human *HDAC3-sgRNA1*, 5’-TTCCCACTGCTGCCAAAAGA-3’ and 5’-TTGTGGGATGAGGGGAATGC-3’ for human *HDAC3-sgRNA2*. Cells showed clear gene editing efficiency in desired targeting locus in each gene were expanded and subjected to HLC differentiation and functional analysis.

Differentiation of hPSCs into HLCs

Differentiation of hPSCs into HLCs was performed following the protocols of Si-Tayeb et al(Si-Tayeb et al., 2010). Briefly, hPSCs were 1) incubated in RPMI-B27 (RPMI-1640 from Invitrogen, 11875093; B27 supplement from Invitrogen, 12587010) medium supplemented with recombinant activin A (100 ng/mL, PeproTech, AF-120-14E) and LY-294002 (5 μM, Selleck, S1105) for 3 or 4 days to obtain definitive endoderm; 2) RPMI-B27 supplemented with BMP4 (20 ng/mL, PeproTech, 120-05) and FGF2 (5 ng/mL, PeproTech, AF-100-18B) and 0.5% DMSO for 5 days to get hepatoblasts; 3) RPMI-B27 supplemented with HGF (20 ng/mL, PeproTech, 100-39) and 0.5% DMSO for 5 days to get immature hepatocytes; and 4) HCM Hepatocyte Culture Medium (Lonza, CC-3198) supplemented with HGF (20 ng/mL), Oncostatin M (20 ng/mL, PeproTech, 300-10), dexamethasone (100 nM, Sigma, D4902) and 0.5% DMSO for 7-9 days to get mature HLCs.

ELISAs, immunocytochemistry and western blot analysis

For ELISA experiments, supernatant from differentiated HLCs or primary hepatocytes were collected at the end of differentiation (for HLCs) or on day 7 after *in vitro* culture (for primary hepatocytes) to determine the concentration of human albumin (Abcam, ab108788), human AFP (Abcam, ab193765), mouse albumin (Abcam, ab108792), respectively. Total RNA amount was used for normalization.

For immunocytochemistry analysis, differentiated HLCs or primary hepatocytes at indicated days were fixed, permeabilized with 0.1% Triton-X-100, and counterstained for albumin (Abcam, ab207327) as indicated. Nuclei were visualized with Hoechst stain (Invitrogen, H3570) and images were taken by microscopy (OLYMPUS, IX73).

For western blot analysis of *HDAC3* KO cells, differentiated HLCs treated with *HDAC3* CRISPRs or control vectors were collected. Total proteins were extracted and subjected to regular western procedure against HDAC3 (Abcam, ab32369) and tubulin (Sigma, T6557). For western blot analysis of samples in different stages during hepatic differentiation, cells were fractionated to get cytosol and nuclear sections (Thermo Scientific, 78835) and subjected to analysis with the following antibodies: HDAC3 (Abcam, ab32369), albumin (Abclonal, A0353), H3K9ac (Abcam, ab10812), H3K27ac (Abcam, ab4729), H3 (Abcam, ab10799) and tubulin (Sigma, T6557).

Quantitative RT-PCR

Total RNA was isolated from HLCs, reverse transcription and real-time PCR were performed as described[8] using standard methods. The sequences of primers were as following: 5’-GCACACTTTCTGAGAAGGAGAG-3’ and 5’-CACTTCTCTACAAAAGCTGCG-3’ for human *ALB* gene; 5’-TCAGTGAGGACAAACTATTGGC-3’ and 5’-GGGTTTACTGGAGTCATTTCATG-3’ for human *AFP* gene; 5’-GGAACCTATGATCTGAAGAGCG-3’ and 5’-TGGTCAGCACAGCCTTATG-3’ for human *A1AT* gene; 5’-GAGCAGAAATTTGTCCAGCAC-3’ and 5’-CCTCCAGTTCTTGAAGCCC-3’ for human *ASGPR* gene; 5’-TTCACCGTGACCCAAAGTAC-3’ and 5’-TGAGAGCAAACCTCATGCC-3’ for human *CYP3A4* gene; 5’-TTTCAGTCAAGGACCACGTC-3’ and 5’-GAGCACTCCAGCAAAGAGG-3’ for human *GLUT2* gene; 5’-GTCCCCAAAGAGTTTAAAGCTG-3’ and 5’-ACAGTCTTCAGTTGCTCCG-3’ for mouse *Alb* gene ; 5’-GCCCCTTAAACACTTGGATTG-3’ and 5’-CCCATGTCCGTACCAGTTATC-3’ for mouse *Asgpr* gene ; 5’-TGTGTCCCAGTTTGAATCCTC-3’ and 5’-GTTATCCCAGAAGTCCCGAG-3’ for mouse *Apoa1* gene ; 5’-AAAACAGTTGGAGCAAAGGC-3’ and 5’-CAAGGGTCCCAGCTTTTCTAG -3’ for mouse *Apoa5* gene ; 5’-AGGAGTCCGATATAGCTGTGG-3’ and 5’-CTCACGACTCAATAGCTGGAG-3’ for mouse *Apoc3* gene ; 5’-TTGGCAAGGTGATGGAAGAG-3’ and 5’-TCTCTGTAAGATAGGCCTCCC -3’ for mouse *Arg1* gene ; 5’-CAATCCCTTGGTTCATGGTTG-3’ and 5’-AGGAAGTCCGCAATGTACTG-3’ for mouse *Glut2* gene ; 5’-GCCAACTTTGCTCGGAATG-3’ and 5’-TCCACTTCCTTGTCTTTCAGC-3’ for mouse *Ces1g* gene ; 5’-CGGCTACCACATCCAAGGAA-3’ and 5’-GCTGGAATTACCGCGGCT-3’ for 18S RNA.

Plasmids and Co-immunoprecipitation

The plasmids of HA-HNF4 and HDAC3-Flag were constructed by standard molecular cloning techniques. The *HNF4* gene was amplified and cloned into the pCDH vector (System Biosciences, CD527A-1) with an HA-tag inserted to 5’ of the cDNA. The *HDAC3* gene was constructed into pcDNA3.0 vector with a FLAG-tag at 3’ of

the cDNA. These two plasmids were co-transfected into HEK293T cells using Lipofectamine 2000 (Invitrogen, 11668019) following the manual protocol. Forty-eight hours after transfection, HEK293T cells were lysed with the RIPA buffer (Millipore, 20-188). The whole cell lysate was then immunoprecipitated with anti-M2 beads (Sigma, A2220) at 4 ℃ overnight. Beads were then washed three times with the RIPA buffer, and bound HNF4 was immunoblotted with an anti-HA antibody (Cell Signaling Technology, 3724).

Dual-Luciferase reporter assay

The human *ALB* promoter/enhancer region containing HNF4 binding sites (-7264～-5992 from *ALB* TSS) was amplified from iPSC genomic DNA and cloned into the pGL3-basic luciferase vector (Promega, E1751). Luciferase assays were carried out by transiently transfecting HEK293T cells using Lipofectamine 2000. Briefly, HEK293T cells were seeded into 24-well plates and transfected with 100 ng of indicated luciferase reporter constructs and 10 ng pRL-TK in each well together with 200 ng HNF4 and/or 200 ng HDAC3. The empty vector pCDH was selected to maintain equal amounts of DNA among wells. Cells were lysed 24 hours post-transfection and analyzed for luciferase activity with the dual-luciferase assay Kit (Promega, E1910) according to the manufacturer's protocol.

Chromatin immunoprecipitation

ChIP assays were carried out using the SimpleChIP® Enzymatic ChIP Kit (Cell Signaling Technology, 9002) following the manufacturer's protocol. Briefly, HLCs (2×10^7cells) treated with CI-994 or DMSO were fixed with 1% formaldehyde for 10min at room temperature and quenched with glycine for 5 min. The cells were then lysed with the lysis buffer. Chromatin was later digested at 37 ℃ for 10min to generate fragments with an average length of 150-900 bp. The samples were then sonicated on ice and supernatant was transferred to a new tube. 2% supernatant was saved for input at this stage, and the remnant was subjected to immunoprecipitation at 4 ℃ overnight with the following antibodies, respectively: anti-HNF4a (Abcam, ab181604), anti-H3K9ac (Abcam, ab10799), anti-H3K27ac (Abcam, ab4729) and IgG (Cell Signaling Technology, 2729) as control. Each reaction was then incubated with protein G agarose beads for another 2 hours at 4 ℃. After standard washes, elution buffer was added to all immunoprecipitation samples and the input samples. DNA from each sample was then purified for real-time PCR. Primers used in the real-time PCR were as following: 5'- AATGGGAATTAGTACTGGTTTGGGA-3', and 5'-ACAGTCATTGCCCAGAATGTTAC-3' for anti-HNF4; 5'-CTGAACAGCCAAACAGAGATTCC-3', and 5'-ACCATGGGAATCCTATCCCACTA-3' for anti-H3K9ac and anti-H3K27ac. Fold enrichment was then calculated as: Percent Input = $2\% \times 2^{(C[T]\ 2\%\text{Input Sample} - C[T]\ \text{IP Sample})}$; C[T] = CT = Threshold cycle of PCR reaction.

RNA-seq analysis

Total RNA from differentiated HLCs treated with CI-994 or DMSO control was extracted and subjected to quality assessment. In total six samples were prepared with three replicates in either group and sequencing was performed in WuXi Apptec Co. Ltd (Shanghai). For data analysis, the raw RNA-seq reads were aligned to human genome (hg19) by STAR. The number of reads for each RefSeq gene was counted by HTSeq(Pruitt et al., 2007). Differentially expressed genes were then identified by DEseq(Anders and Huber, 2010) by using fold change ≥ 2 and FDR ≤ 0.01 as cutoff. GO term and pathway annotations were downloaded from Gene Ontology website. GO enrichment analysis were performed by homemade scripts of R and Python scripts using Fisher's exact test.

Statistical analysis

The unpaired, two-tailed Student's t test was used for experiments with two groups, and one-way ANOVA test and post-hoc Bonferronic multiple-comparison test was used for experiments that contained more than two groups. All data are represented as means with SEM.

Supplemental References

Anders, S., and Huber, W. (2010). Differential expression analysis for sequence count data. Genome Biol *11*, R106.

Cowan, C.A., Klimanskaya, I., McMahon, J., Atienza, J., Witmyer, J., Zucker, J.P., Wang, S., Morton, C.C., McMahon, A.P., Powers, D., et al. (2004). Derivation of embryonic stem-cell lines from human blastocysts. N Engl J Med 350, 1353-1356.

Dentin, R., Pegorier, J.P., Benhamed, F., Foufelle, F., Ferre, P., Fauveau, V., Magnuson, M.A., Girard, J., and Postic, C. (2004). Hepatic glucokinase is required for the synergistic action of ChREBP and SREBP-1c on glycolytic and lipogenic gene expression. J Biol Chem *279*, 20314-20326.

Ding, Q., Lee, Y.K., Schaefer, E.A., Peters, D.T., Veres, A., Kim, K., Kuperwasser, N., Motola, D.L., Meissner,

T.B., Hendriks, W.T., *et al.* (2013). A TALEN genome-editing system for generating human stem cell-based disease models. Cell Stem Cell *12*, 238-251.
Langmead, B., Trapnell, C., Pop, M., and Salzberg, S.L. (2009). Ultrafast and memory-efficient alignment of short DNA sequences to the human genome. Genome Biol *10*, R25.
Li, W., Xu, H., Xiao, T., Cong, L., Love, M.I., Zhang, F., Irizarry, R.A., Liu, J.S., Brown, M., and Liu, X.S. (2014). MAGeCK enables robust identification of essential genes from genome-scale CRISPR/Cas9 knockout screens. Genome Biol *15*, 554.
Pruitt, K.D., Tatusova, T., and Maglott, D.R. (2007). NCBI reference sequences (RefSeq): a curated non-redundant sequence database of genomes, transcripts and proteins. Nucleic Acids Res *35*, D61-65.
Si-Tayeb, K., Noto, F.K., Nagaoka, M., Li, J., Battle, M.A., Duris, C., North, P.E., Dalton, S., and Duncan, S.A. (2010). Highly efficient generation of human hepatocyte-like cells from induced pluripotent stem cells. Hepatology *51*, 297-305.
