# Supplementary material for: Genetic and Chemical Screenings Identify HDAC3 as a Key Regulator in Hepatic Differentiation of Human Pluripotent Stem Cells
Source: Stem Cell Reports. 2018 May 31;11(1):22–31. doi: 10.1016/j.stemcr.2018.05.001 (PMC6066908; doi:10.1016/j.stemcr.2018.05.001)
Supplement: Document S2. Article plus Supplemental Information [file mmc2.pdf]

Stem Cell Reports
Report

OPEN ACCESS

Genetic and Chemical Screenings Identify HDAC3 as a Key Regulator in Hepatic Differentiation of Human Pluripotent Stem Cells

Shuang Li,[1] Mushan Li,[2] Xiaojian Liu,[1] Yuanyuan Yang,[1] Yuda Wei,[1] Yanhao Chen,[1] Yan Qiu,[1] Tingting Zhou,[1] Zhuanghui Feng,[1] Danjun Ma,[3] Jing Fang,[1] Hao Ying,[1] Hui Wang,[1,4] Kiran Musunuru,[5] Zhen Shao,[2,*] Yongxu Zhao,[1,3,*] and Qiurong Ding[1,*]
[1]CAS Key Laboratory of Nutrition, Metabolism and Food Safety, Shanghai Institute of Nutrition and Health, Shanghai Institutes for Biological Sciences, University of Chinese Academy of Sciences, Chinese Academy of Sciences, Shanghai 200031, P. R. China
[2]CAS Key Laboratory of Computational Biology, CAS-MPG Partner Institute for Computational Biology, Shanghai Institute of Nutrition and Health, Shanghai Institutes for Biological Sciences, Chinese Academy of Sciences, Shanghai 200031, P. R. China
[3]College of Mechanical Engineering, Dongguan University of Technology, Dongguan, Guangdong 523808, P. R. China
[4]School of Public Health, Shanghai Jiao Tong University School of Medicine, Shanghai 200025, P. R. China
[5]Cardiovascular Institute, Department of Medicine, Department of Genetics, Perelman School of Medicine at the University of Pennsylvania, Philadelphia, PA 19104, USA
*Correspondence: shaozhen@picb.ac.cn (Z.S.), yxzhao@sibs.ac.cn (Y.Z.), qrding@sibs.ac.cn (Q.D.)
https://doi.org/10.1016/j.stemcr.2018.05.001

SUMMARY

Hepatocyte-like cells (HLCs) derived from human pluripotent stem cells (hPSCs) offer a promising cell resource for disease modeling and transplantation. However, differentiated HLCs exhibit an immature phenotype and comprise a heterogeneous population. Thus, a better understanding of HLC differentiation will improve the likelihood of future application. Here, by taking advantage of CRISPR-Cas9-based genome-wide screening technology and a high-throughput hPSC screening platform with a reporter readout, we identified several potential genetic regulators of HLC differentiation. By using a chemical screening approach within our platform, we also identified compounds that can further promote HLC differentiation and preserve the characteristics of *in vitro* cultured primary hepatocytes. Remarkably, both screenings identified histone deacetylase 3 (HDAC3) as a key regulator in hepatic differentiation. Mechanistically, HDAC3 formed a complex with liver transcriptional factors, e.g., HNF4, and co-regulated the transcriptional program during hepatic differentiation. This study highlights a broadly useful approach for studying and optimizing hPSC differentiation.

INTRODUCTION

Human pluripotent stem cells (hPSCs) hold great promise as an attractive resource of human somatic cells, due to their ability to self-renew and, theoretically, to differentiate into any of the myriad of somatic cell types in the human body. In practice, the ability to differentiate into a desired cell type often depends on the availability of an efficient protocol. Stepwise protocols using defined factors have been established years ago for the differentiation from hPSCs toward human hepatocytes (Si-Tayeb et al., 2010; Song et al., 2009; Sullivan et al., 2010). However, the resulting hepatocytes exhibit an immature hepatic phenotype (e.g., express fetal markers such as alpha fetoprotein) and remain a heterogeneous population. In addition, substantial variation in differentiation efficiencies has been observed among different hPSC lines. Considerable effort has later been devoted to studying the directed differentiation process or screening for compounds for enhanced maturation of HLCs (Cheng et al., 2012; Li et al., 2017; Loh et al., 2014; Shan et al., 2013). While many of the improvements to the hepatic differentiation protocol are based on the knowledge acquired in understanding signal pathways that control embryonic lineage bifurcations, none of the existing studies tackled the differentiation problems through an unbiased genetic screening approach. With recent advances in genome editing technologies, especially the clustered regularly interspaced short palindromic repeats (CRISPR)/CRISPR-associated (Cas) system, genome-wide genetic screening becomes a high-throughput, low-cost platform that enables comprehensive study of regulators in a biological process (Shalem et al., 2015). In the present study, we devised a reporter system and applied a CRISPR/Cas9-based genetic screening approach to identify potential regulators in hepatic lineage determination. Based on the results from the genetic screening, we also performed a targeted, small-scale chemical screening. We have identified several regulators in hepatic differentiation and an efficient small molecule that can improve the differentiation efficiency. Our study also demonstrated a broadly useful approach for studying hPSC differentiation.

RESULTS

Generation of the *ALB-Venus* Reporter Line

Albumin is regarded as a molecular marker of hepatocytes that can reflect cell maturation. To perform screenings for regulators in HLC differentiation, we developed a reporter

This is an open access article under the CC BY license (http://creativecommons.org/licenses/by/4.0/).

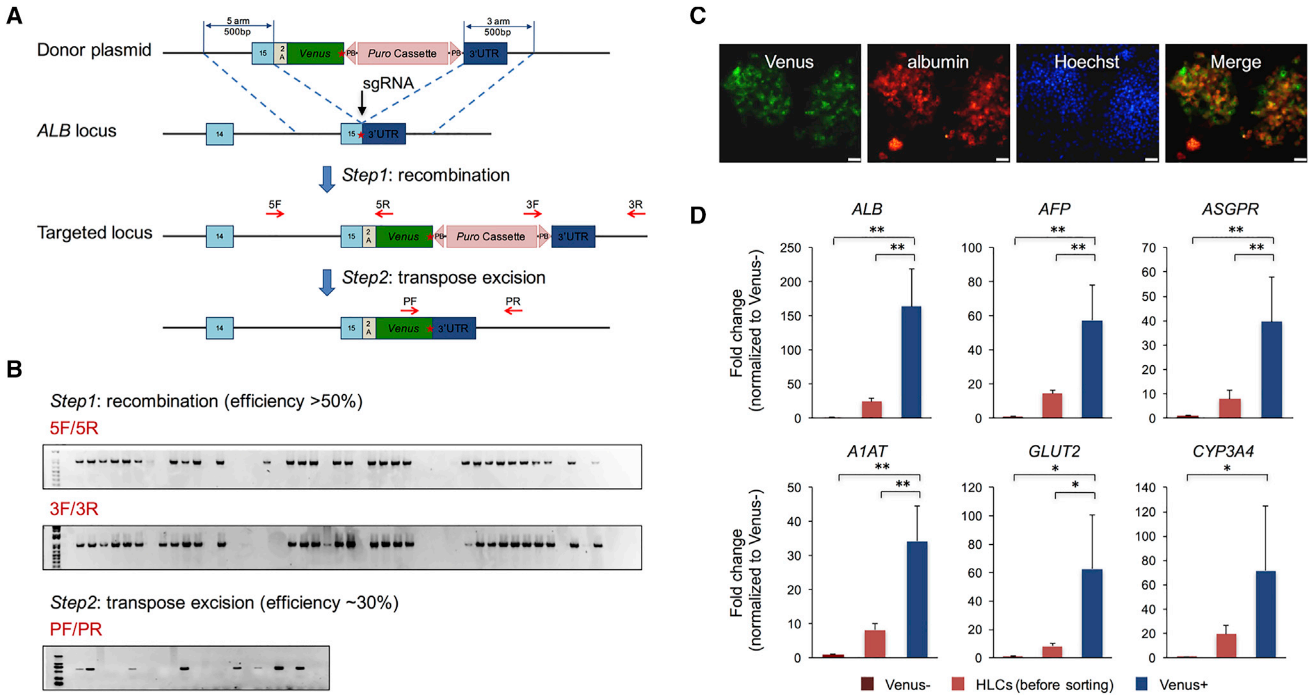

Figure 1. Generation of the *ALB-Venus* Reporter Line

(A) Schematic view of the targeting strategy. The red star indicates the stop codon of the *ALB* gene. Arrows indicate screening primers used for genotyping of individual clones.

(B) Representative PCR screening results in both recombination and transposon excision steps.

(C) Albumin staining of HLCs derived from one positive hPSC clone (scale bar, 50 μm).

(D) Gene expression analysis of different cell populations from before or after FACS of HLCs derived from one positive hPSC clone. n = 3 independent experiments.

Data are represented as means with SEM. *p < 0.05, **p < 0.01.

hPSC line with a T2A-Venus cassette knocked in before the stop codon of the endogenous *ALB* gene, termed the *ALB-Venus* reporter line (Figure 1A). A previously established induced pluripotent stem cell line, DiPS 1016 SevA (called 1016 for short), with relatively poor HLC differentiation efficiency, was chosen as the parental hPSC line. We designed and screened for an efficient single guide RNA (sgRNA) targeting the site of the *ALB* stop codon. CRISPR-Cas9 homology-directed repair with a donor template, followed by transposon excision yielded cells with one or two knocked-in alleles at a relatively high efficiency (more than 50% at step 1 and ~30% at step 2) (Figures 1A and 1B). To validate the obtained reporter cell line, we differentiated hPSCs into HLCs using an adapted protocol (Ding et al., 2013; Si-Tayeb et al., 2010). Image analysis of HLCs displayed a very high degree of overlap between the Venus and albumin signals (Figure 1C). HLCs were also sorted based on the intensity of Venus expression to compare three cell populations: Venus–, Venus+, and presorted HLCs; and Venus+ cells had significantly higher expression levels of several hepatic markers compared with either presorted HLCs or Venus– HLCs (Figure 1D).

Genetic Screenings Reveal that HDAC3 Is Involved in the Regulation of HLC Differentiation

Using the *ALB-Venus* reporter cell line, we set up a genome-wide genetic screening by using the GeCKO library (Shalem et al., 2014) (Figure 2A). hPSCs were transduced by the GeCKO library and differentiated toward HLCs. HLCs were then sorted to obtain high Venus+ (top 5%) and Venus– (bottom 5%) populations. Two other cell groups, presorted HLCs and hPSCs, were also collected. Each population of cells was subjected to genomic DNA isolation and deep sequencing of integrated sgRNAs. Candidate genes were initially screened for significant enrichment of sgRNAs in the Venus+ population compared with the presorted HLCs population. The sgRNA counts in the other two control groups, the Venus– HLCs and hPSCs, were also referenced when winnowing the list of candidate genes. Five genes stood out as top candidates: *TIMP3*, *RAB3-GAP1*, *ATG7*, *RPS6KA2*, and *HDAC3* (Figures S1A and S1B). To validate these candidate genes, individual gene-knockout hPSCs were generated by CRISPR-Cas9-mediated targeting and subjected to HLC differentiation. Among the five tested candidate genes, $ATG7^{-/-}$, $RPS6KA2^{-/-}$, and

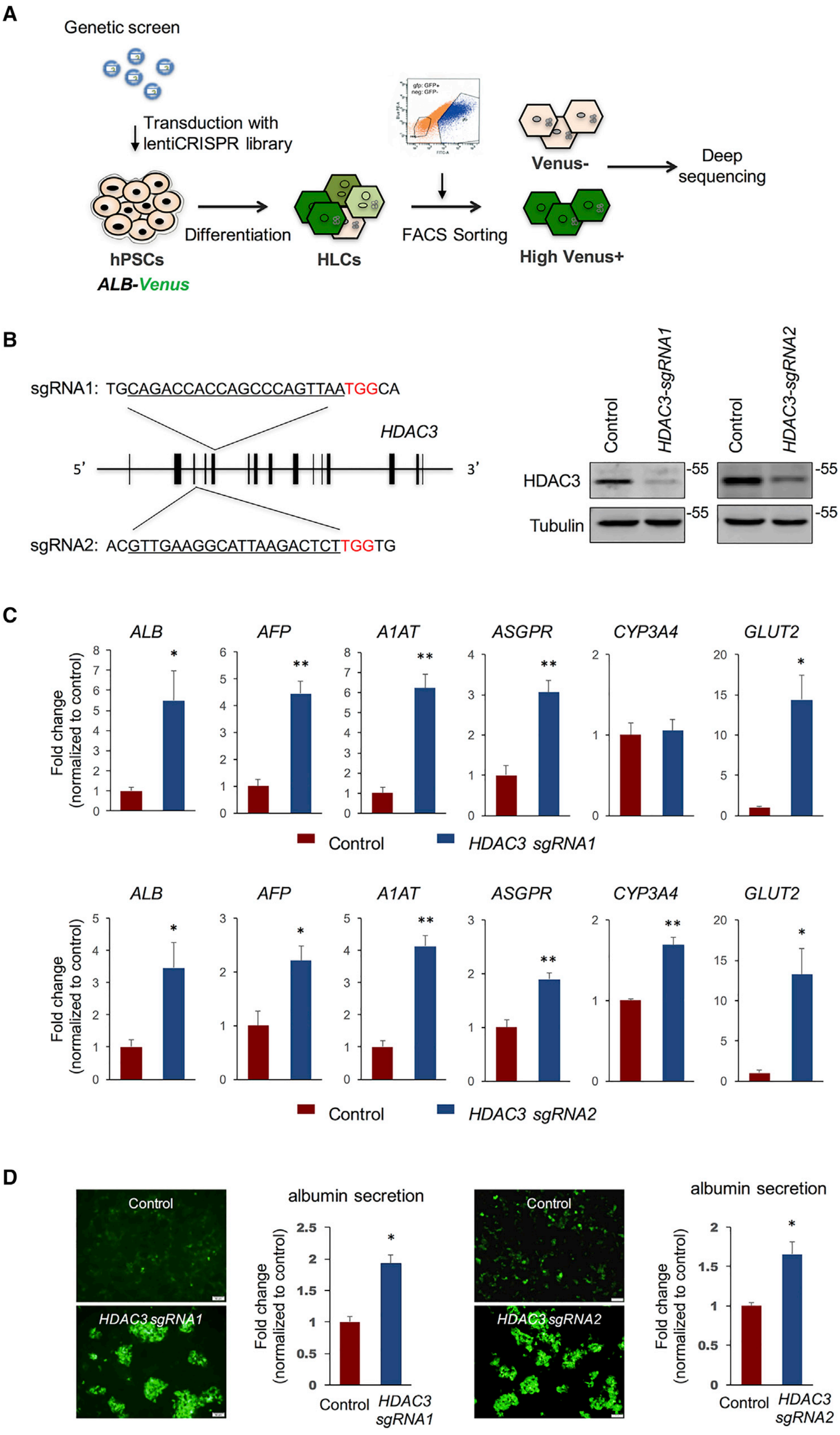

(legend on next page)

HDAC3$^{-/-}$ hPSCs displayed significantly increased differentiation efficiency compared with control cells (Figures 2B–2D, S1C, and S1D). On the other hand, we did not observe obvious changes in HLCs from *TIMP3*$^{-/-}$ and *RAB3GAP1*$^{-/-}$ hPSCs (data not shown).

Chemical Screenings Identify that CI-994 Improves HLC Differentiation

Of the three genes we successfully validated, *ATG7* encodes autophagy-related protein 7 (Ohsumi, 2001); *RPS6KA2* encodes ribosomal protein S6 kinase alpha-2, a member of the ribosomal S6 kinase family of serine and threonine kinases that signals downstream of the mitogen-activated protein kinase pathway (Bignone et al., 2007); and *HDAC3* encodes histone deacetylase 3, a class I histone deacetylase that is a core component of nuclear receptor corepressor complexes and was previously found to regulate liver triglyceride homeostasis (Papazyan et al., 2016; Sun et al., 2012) and be critical for maintaining genome stability in liver cells (Bhaskara et al., 2010). With the goal of directly translating these findings into improvements of HLC differentiation protocol, we next assessed whether small molecules can reproduce the effects caused by reduced expression of these genes. We first tested two autophagy inhibitors (wortmannin and chloroquine), as well as an inhibitor of ribosomal protein S6 kinase (BID1870) (Sapkota et al., 2007). We noticed slightly improved differentiation of HLCs by wortmannin treatment (Figure S2A), whereas no significant improvement by chloroquine or BID1870 treatment (data not shown).

The involvement of *HDAC3* in HLC differentiation suggested that inhibition of histone deacetylation can improve HLC differentiation. Rather than testing individual compounds, we next devised a chemical screening platform with the *ALB-Venus* reporter line. As HLCs are typically stacked in multiple layers in a culture dish at the end of more than 20 days of differentiation, complicating the ability to perform image-based screening, we split the cells at the stage of immature hepatocytes and re-plated in a single layer in 96-well plates for further maturation and chemical screening (Figure 3A). In total, 43 epigenetic regulators were screened; treatment with CI-994, which represents a selective inhibitor of class I HDACs (Undevia et al., 2004), resulted in a dramatic increase in Venus expression intensity as well as percentage of positive cells; whereas an inhibitor to histone acetyltransferases (HAT), Garcinol, resulted in the lowest Venus expression (Figure S2B).

Further analysis showed that HLCs treated with CI-994 displayed significantly higher expression levels of several hepatic markers, as well as reduced expression of the fetal liver marker *AFP*, compared with control cells (Figures 3B, S3A, and S3B). Primary human hepatocytes (PHHs) were also included for comparison. As primary hepatocytes lose hepatic characteristics rapidly when cultured *in vitro*, both freshly isolated cells (PHH-Day0) and cells after culturing *in vitro* for 2 days (PHH-Day2) were included. Results indicated that after CI-994 treatment, HLCs remained immature when compared with PHH-day0; however, better performed PHH-day2 in some aspects, such as increased albumin secretion (Figure 3B). In the meantime, CI-994 treatment outperformed *HDAC3*$^{-/-}$ hPSCs, as reflected by reduced AFP expression, we thus suspect that HDAC1 and 2 may also involve in hepatic differentiation, albeit they were not picked up by the genetic screening. Possible reasons may lie in that the screening was initially designed to test effects of individual genes, and there may exist compensation effects between HDAC1 and 2. RNA sequencing (RNA-seq) analysis was next performed on HLCs. CI-994 treatment led to 182 upregulated genes and 34 downregulated genes with a cutoff of false discovery rate (FDR) $\leq$0.01 and fold change $\geq$2. Several GO terms relevant to hepatic functions were significantly enriched in upregulated genes (Figure 3C). No GO term showed significant enrichment in downregulated genes.

CI-994 Treatment Also Delays the Loss of Hepatocyte Characteristics of Primary Hepatocytes Cultured *In Vitro*

Besides the differentiation of HLCs from hPSCs, a complementary approach to studying hepatocyte function *in vitro* is to isolate and culture primary hepatocytes. However, primary hepatocytes are known to rapidly de-differentiate and lose hepatic characteristics *in vitro*, although several culture conditions that can maintain hepatocytes in

Figure 2. Genetic Screenings Reveal that *HDAC3* Is Involved in the Regulation of HLC Differentiation
(A) Schematic view of the genetic screening strategy. See also Figures S1A and S1B.
(B) SgRNA sequences targeting human *HDAC3* gene (underlined part, left); western blot analysis of HDAC3 protein in CRISPR-Cas9-treated HLCs and control cells.
(C) Gene expression analysis of CRISPR-Cas9-treated HLCs and control cells from three independent experiments.
(D) Representative images of Venus intensity in HLCs treated with CRISPR-*HDAC3* and control viruses (scale bar, 50 μm); albumin mass measured by ELISA in media collected from HLCs treated with CRISPR-*HDAC3* or control viruses, normalized to mean levels of control group (n = 3 independent experiments).
Data are represented as means with SEM. *p < 0.05, **p < 0.01. See also Figures S1C and S1D.

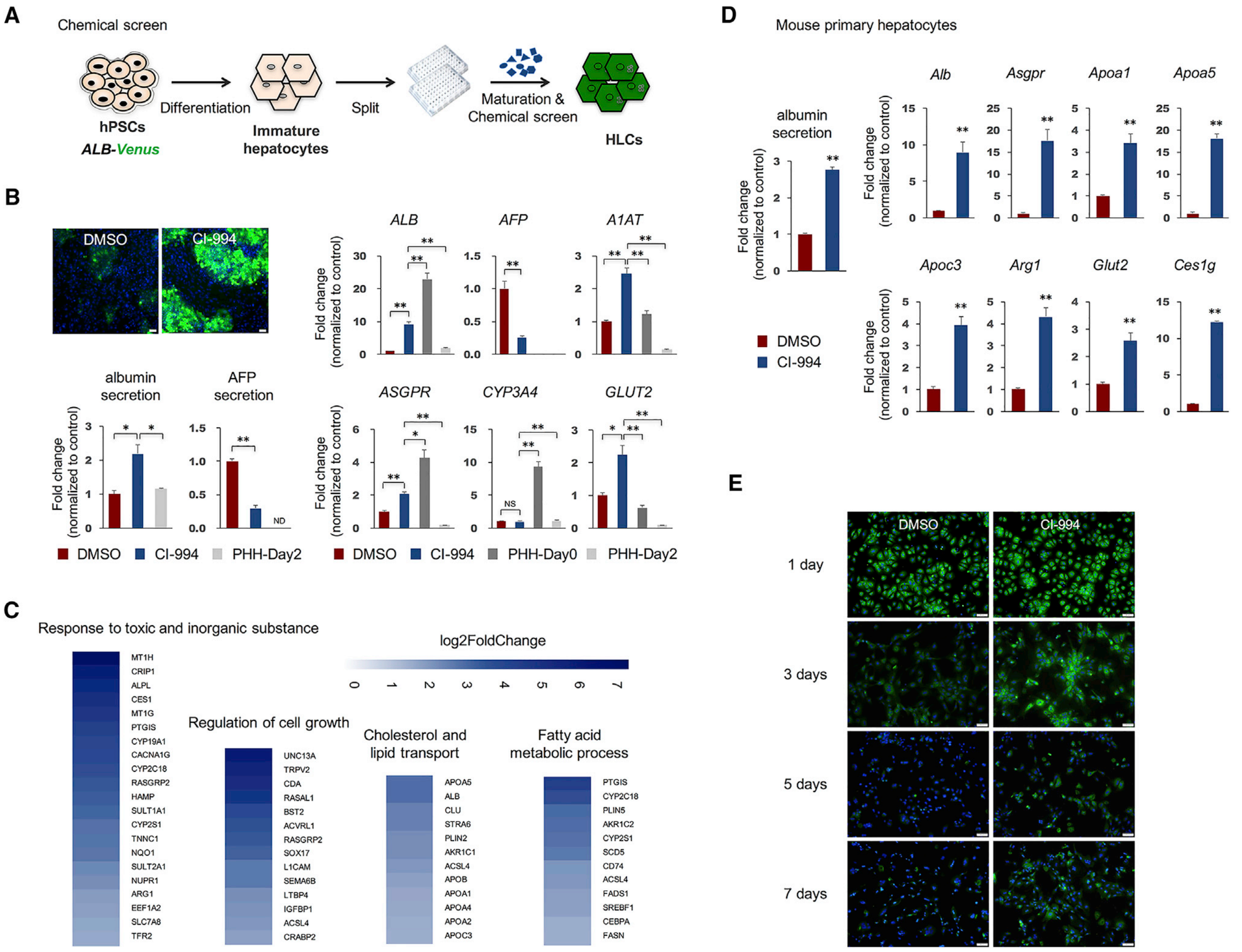

Figure 3. Chemical Screenings Identify that CI-994 Improves HLC Differentiation and Delays the Loss of Hepatocyte Characteristics of Primary Hepatocytes Cultured *In Vitro*

(A) Schematic view of the chemical screening strategy.

(B) Representative images of Venus intensity in HLCs treated with 10 μM CI-994 or DMSO control (scale bar, 50 μm) (above left); albumin and AFP mass measured by ELISA in media collected from HLCs treated with 10 μM CI-994 or DMSO control and PHH-day2, normalized to mean levels of control group (below left); gene expression analysis of 10 μM CI-994-treated HLCs, control HLCs, PHH-day0 and PHH-day2 (right). n = 3 independent experiments. ND, not determined; NS, not significant. See also Figures S2, S3A, and S3B.

(C) RNA-seq analysis of 10 μM CI-994-treated HLCs and control cells. Shown are genes upregulated in CI-994-treated cells compared with control cells.

(D) Albumin mass measured by ELISA in media collected from primary mouse hepatocytes cultured *in vitro* and treated with 10 μM CI-994 or DMSO control for 7 days, normalized to mean levels of control group (left); gene expression analysis of above cell groups (right). n = 3 independent experiments.

(E) Representative albumin staining of primary hepatocytes treated with 10 μM CI-994 or DMSO control for indicated days (scale bar, 50 μm).

Data are represented as means with SEM. *p < 0.05, **p < 0.01. See also Figure S3C.

culture for a limited time period have been reported (Block et al., 1996; Mitaka et al., 1991; Mizuguchi et al., 2001; Richman et al., 1976). We next asked whether CI-994 can also delay the de-differentiation of primary hepatocytes *in vitro*. Indeed, we found that primary hepatocytes treated with CI-994 better maintained their morphology and hepatic gene expression compared with control cells (Figures 3D, 3E, and S3C). These results suggest that CI-994 might be useful for the maintenance of primary hepatocytes in culture.

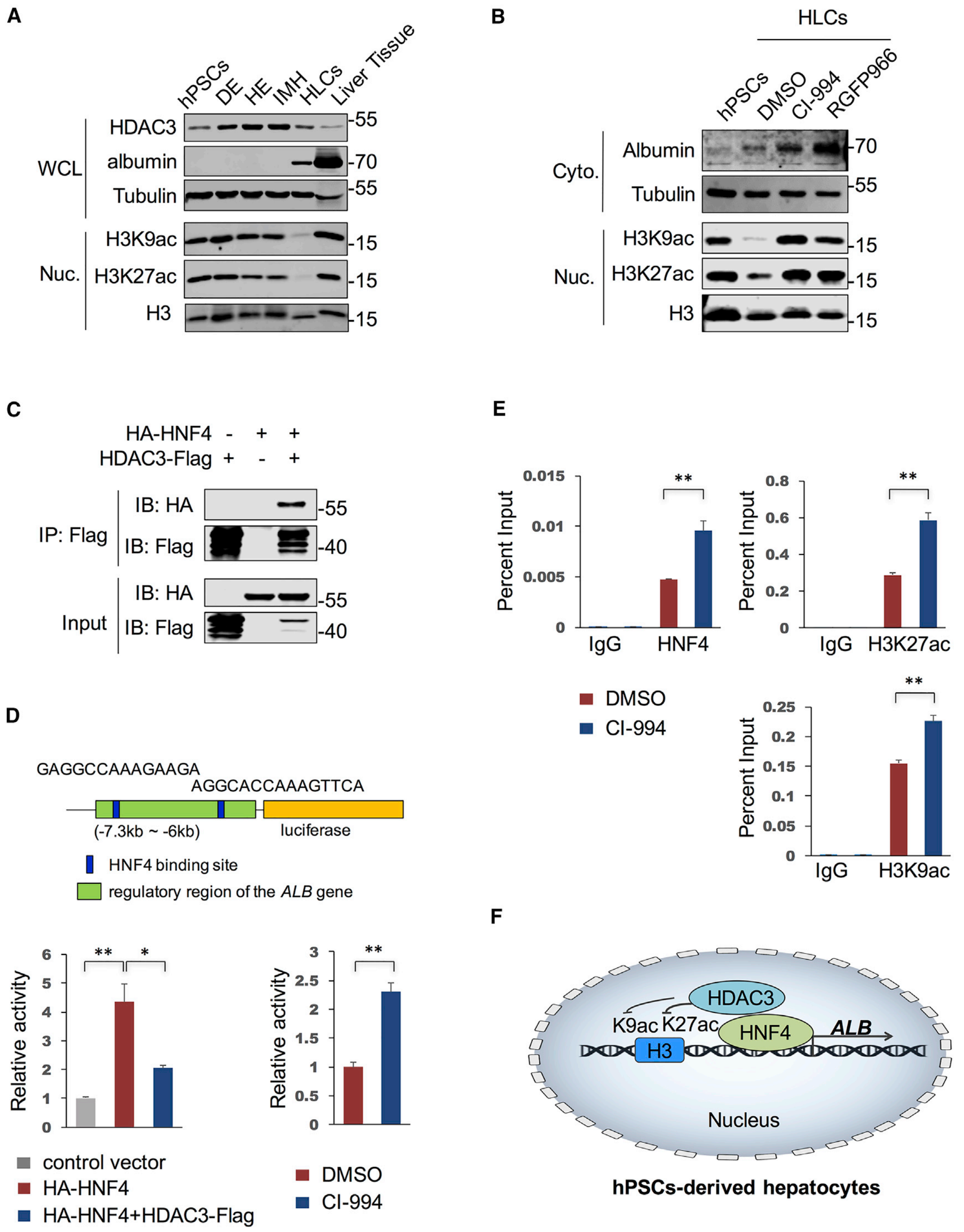

Figure 4. HDAC3 Coordinates with HNF4 in Regulating *ALB* Expression in HLCs

(A) Western blot analysis of cells at different stages in hepatic differentiation and primary human liver tissue. WCL, whole cell lysates; DE, definitive endoderm; HE, hepatic endoderm; IMH, immature hepatocytes.

(B) Western blot analysis of hPSCs and HLCs treated with CI-994 (5 μM) or RGFP966 (10 μM) or DMSO as control. The cytosol (Cyto.) and nuclear (Nuc.) sections of each sample in (A) and (B) were separated for analysis.

(C) Co-immunoprecipitation analysis of HNF4 and HDAC3 in HEK293T cells.

(legend continued on next page)

HDAC3 Coordinates with HNF4 in Regulating *ALB* Expression in HLCs

Both the genetic and chemical screenings suggest that inhibition of class I HDACs, specifically HDAC3, improves hepatic differentiation of hPSCs. We noticed a dynamic change of HDAC3 expression levels along the hepatic differentiation process and a dramatic decrease of both H3K9ac and H3K27ac nuclear signals at HLC stage, in comparison with signals in cells from other stages as well as in the primary human liver tissue (Figure 4A). Treatment with CI-994 or RGFP966—a specific inhibitor to HDAC3—reversed the levels of both H3K9ac and H3K27ac, and enhanced the expression of albumin in HLC stage (Figure 4B). It is well known that acetylation of H3K9 and H3K27 mark actively transcribed regions in genome (Lawrence et al., 2016), which can be deacetylated by HDACs, resulting in reduced local gene transcription (Allis and Jenuwein, 2016). These results suggest that the low histone acetylation level in HLC stage as part of the causation of compromised hepatic gene expression in HLCs compared to human primary liver tissue.

Hepatocyte differentiation is dependent on activation of a series of hepatic transcription factors, such as HNF4 and CEBP (Li et al., 2000; Nagy et al., 1994). Since HNF4 was found prominently at co-occupied sites with HDAC3 on the mouse liver genome (Armour et al., 2017; Papazyan et al., 2016), we next investigated whether HDAC3 regulated hepatic gene expression, e.g., albumin, through interacting with HNF4. Indeed, our results confirmed the interaction between HNF4 and HDAC3 (Figure 4C). We further assessed whether HDAC3 and HNF4 co-regulate the albumin expression. Several HNF4 binding sites were predicted in the enhancer region of the *ALB* gene (−7.3 kb to −6 kb from transcription start site [TSS]). We cloned this regulatory region into a luciferase reporter plasmid. We found that HNF4 overexpression significantly enhanced the luciferase signal, which could be attenuated by HDAC3 overexpression. And CI-994 treatment increased the luciferase signal (Figure 4D). Chromatin immunoprecipitation (ChIP)-qPCR analysis further confirmed that CI-994 treatment also increased HNF4 binding activity and both signals of H3K9ac and H3K27ac in the *ALB* enhancer region in HLCs (Figure 4E). Altogether, these results indicated a working mechanism that the epigenetic regulator HDAC3 coordinates with hepatic transcription factors, e.g., HNF4, in regulating the expression of lineage-specific genes during hepatic differentiation of hPSCs (Figure 4F). It is interesting to read in a recent publication that HDAC3 organizes heterochromatin at the nuclear lamina and therefore regulates the cardiac differentiation of mouse embryonic stem cells (mESCs) (Poleshko et al., 2017). A deeper understanding to the role of HDAC3 in hepatic differentiation warrants further investigation.

DISCUSSION

It remains a general problem in the stem cell field that somatic cells derived from hPSCs are all at a relatively immature stage, and many *in vitro* differentiation systems require further optimization. We addressed this problem by developing a cellular platform that could be applied to high-throughput screening for both genetic factors and chemical compounds that improve differentiation. This approach can readily be adapted to improve other differentiation protocols. We also note several limitations of the approach. (1) Reporter cell lines only reflect a limited part of the functionality of cells. For example, the *ALB-Venus* reporter line used in our study reflects only the expression of albumin protein, which does not necessarily represent fully mature hepatocytes. Indeed, in our study, HLCs with either genetic ablation of HDAC3 or CI-994 treatment showed significantly increased *ALB* expression; however, remained at a relatively immature stage with high *AFP* expression and low CYP3A4 expression compared with PHHs. Dual- or multiple-reporter cell lines (e.g., *CYP3A4* as a positive reporter, *AFP* as a negative reporter) could potentially be used to better identify drivers of cell maturity. (2) As most terminal differentiated cells lack the ability to proliferate, the selection step (e.g., the fluorescence activating cell sorting [FACS] step in our study) is less able to enrich for the desired sgRNAs compared with studies in which enhancement of proliferation or acquisition of drug resistance is the desired outcome (Shalem et al., 2014). Because of this, studies with a larger number of cells (or fewer sgRNAs in the library) are needed to identify true positive enriched sgRNAs and eliminate false-negative sgRNAs. (3) Lengthy, multi-step differentiation protocols make it more difficult to screen for factors that will improve cell maturity. For example, in our study, cells were infected with CRISPR

(D) Luciferase reporter assay in HEK293T cells (n = 3 independent experiments). The schematic cartoon of the luciferase reporter construction was shown above.
(E) ChIP analysis with indicated antibodies in HLCs treated with DMSO or CI-994 (5 μM) from three independent experiments. Primers used in qPCR analysis amplified the *ALB* enhancer region (−7 to −6.5 kb).
(F) The schematic diagram of a working mechanism that HDAC3 coordinates with HNF4 in regulating *ALB* expression in HLCs.
Data are represented as means with SEM. $^{*}p < 0.05$, $^{**}p < 0.01$.

lentivirus at the undifferentiated stage and analyzed at the terminal HLC stage, making it harder to identify factors that would have the desired effect when modulated only in a specific stage of differentiation. An inducible CRISPR-Cas9-based platform could potentially define contributors in specific differentiation stages by modulating the time course of gene knockdown (Chen et al., 2015; Gonzalez et al., 2014). These limitations notwithstanding, by combining an hPSC reporter cell line, a CRISPR-Cas9-mediated genetic screening, and a chemical screening, our study highlights a broadly useful approach for the optimization of hPSC differentiation.

EXPERIMENTAL PROCEDURES

Construction of the *ALB-Venus* Reporter Line

Construction of the *ALB-Venus* reporter line was following a similar protocol as described (Ding et al., 2013). Details are given in Supplemental Experimental Procedures.

Genome-wide CRISPR-Cas9 Screenings

In general, the *ALB-Venus* reporter hPSCs were transduced by the CRISPR lentivirus library, followed by hepatic differentiation to obtain HLCs. Cells with high Venus expression (top 5%) were then collected by FACS sorting, and different control groups, including Venus− cells, HLCs, and hPSCs, were also obtained. Genomic DNA from different cell groups was later extracted and subjected to PCR amplification and deep sequencing. sgRNAs and corresponding genes significantly over- or under-represented in the samples of interest were identified. Details are given in Supplemental Experimental Procedures.

Chemical Screenings

The *ALB-Venus* reporter hPSCs were differentiated to get immature hepatocytes. Cells were then split and plated in 96-well plates for further maturation and chemical screening. Cells were then analyzed for Venus expression in a high-throughput platform using High Content Screening (Cellomics ArrayScan VTI; Thermo Fisher Scientific). Details are given in Supplemental Experimental Procedures.

Cell Culture

Cell culture was following a standard protocol. Details are given in Supplemental Experimental Procedures.

Lentivirus Packaging and Generation of CRISPR-Cas9 Knockout Cell Lines

Lentivirus packaging and generation of CRISPR-Cas9 knockout cell lines were following standard methods. Details are given in Supplemental Experimental Procedures.

Differentiation of hPSCs into HLCs

Differentiation was performed following the protocols of Si-Tayeb et al. (2010). Details are given in Supplemental Experimental Procedures.

ELISAs, Immunocytochemistry, and Western Blot Analysis

These procedures were performed using standard methods. Details are given in Supplemental Experimental Procedures.

Quantitative RT-PCR

Quantitative RT-PCR was performed using standard methods. Details and oligonucleotide sequences are given in Supplemental Experimental Procedures.

Plasmid Construction, Co-immunoprecipitation, Dual-Luciferase Reporter Assay, and ChIP

These procedures were performed using standard methods. Details are given in Supplemental Experimental Procedures.

RNA-Seq and Statistical Analysis

RNA-seq and statistical analysis were performed using standard methods. Details are given in Supplemental Experimental Procedures.

ACCESSION NUMBERS

Raw data were deposited in the NCBI Sequence Read Archive with the accession number SRA: SRP139546.

SUPPLEMENTAL INFORMATION

Supplemental Information includes Supplemental Experimental Procedures and three figures and can be found with this article online at https://doi.org/10.1016/j.stemcr.2018.05.001.

AUTHOR CONTRIBUTIONS

S.L., Y.Z., and Q.D. designed the research. S.L. performed all experiments. M.L. performed data analysis for CRISPR screening and RNA-seq experiments. X.L., Y.Y., Y.W., Y.C., Y.Q., T.Z., Y.Z., Z.F., D.M., J.F., H.Y., H.W., and K.M. assisted with either experiments or data analysis. S.L., M.L., H.Y., K.M., Z.S., Y.Z., and Q.D. wrote the manuscript. Z.S., Y.Z., and Q.D. supervised the project.

ACKNOWLEDGMENTS

This work was supported by grants from the National Key R&D Program of China (2017YFA0102800, 2017YFA0103700, 2016YFA0500102, 2016YFC1304905), the Strategic Priority Research Program of the Chinese Academy of Sciences (XDA16030402), the Key Research Program of the Chinese Academy of Sciences (ZDRW-ZS-2017-1, KFZD-SW-213), the National Natural Science Foundation of China (31670829, 81630086, 81427805, 91529305) and the National Youth 1000 Talents Program (Q.D.)

Received: February 1, 2018
Revised: May 1, 2018
Accepted: May 2, 2018
Published: May 31, 2018

REFERENCES

Allis, C.D., and Jenuwein, T. (2016). The molecular hallmarks of epigenetic control. Nat. Rev. Genet. *17*, 487–500.

Armour, S.M., Remsberg, J.R., Damle, M., Sidoli, S., Ho, W.Y., Li, Z., Garcia, B.A., and Lazar, M.A. (2017). An HDAC3-PROX1 corepressor module acts on HNF4alpha to control hepatic triglycerides. Nat. Commun. *8*, 549.

Bhaskara, S., Knutson, S.K., Jiang, G., Chandrasekharan, M.B., Wilson, A.J., Zheng, S., Yenamandra, A., Locke, K., Yuan, J.L., Bonine-Summers, A.R., et al. (2010). Hdac3 is essential for the maintenance of chromatin structure and genome stability. Cancer Cell *18*, 436–447.

Bignone, P.A., Lee, K.Y., Liu, Y., Emilion, G., Finch, J., Soosay, A.E., Charnock, F.M., Beck, S., Dunham, I., Mungall, A.J., et al. (2007). RPS6KA2, a putative tumour suppressor gene at 6q27 in sporadic epithelial ovarian cancer. Oncogene *26*, 683–700.

Block, G.D., Locker, J., Bowen, W.C., Petersen, B.E., Katyal, S., Strom, S.C., Riley, T., Howard, T.A., and Michalopoulos, G.K. (1996). Population expansion, clonal growth, and specific differentiation patterns in primary cultures of hepatocytes induced by HGF/SF, EGF and TGF alpha in a chemically defined (HGM) medium. J. Cell Biol. *132*, 1133–1149.

Chen, Y., Cao, J., Xiong, M., Petersen, A.J., Dong, Y., Tao, Y., Huang, C.T., Du, Z., and Zhang, S.C. (2015). Engineering human stem cell lines with inducible gene knockout using CRISPR/Cas9. Cell Stem Cell *17*, 233–244.

Cheng, X., Ying, L., Lu, L., Galvao, A.M., Mills, J.A., Lin, H.C., Kotton, D.N., Shen, S.S., Nostro, M.C., Choi, J.K., et al. (2012). Self-renewing endodermal progenitor lines generated from human pluripotent stem cells. Cell Stem Cell *10*, 371–384.

Ding, Q., Lee, Y.K., Schaefer, E.A., Peters, D.T., Veres, A., Kim, K., Kuperwasser, N., Motola, D.L., Meissner, T.B., Hendriks, W.T., et al. (2013). A TALEN genome-editing system for generating human stem cell-based disease models. Cell Stem Cell *12*, 238–251.

Gonzalez, F., Zhu, Z., Shi, Z.D., Lelli, K., Verma, N., Li, Q.V., and Huangfu, D. (2014). An iCRISPR platform for rapid, multiplexable, and inducible genome editing in human pluripotent stem cells. Cell Stem Cell *15*, 215–226.

Lawrence, M., Daujat, S., and Schneider, R. (2016). Lateral thinking: how histone modifications regulate gene expression. Trends Genet. *32*, 42–56.

Li, J., Ning, G., and Duncan, S.A. (2000). Mammalian hepatocyte differentiation requires the transcription factor HNF-4alpha. Genes Dev. *14*, 464–474.

Li, Q., Hutchins, A.P., Chen, Y., Li, S., Shan, Y., Liao, B., Zheng, D., Shi, X., Li, Y., Chan, W.Y., et al. (2017). A sequential EMT-MET mechanism drives the differentiation of human embryonic stem cells towards hepatocytes. Nat. Commun. *8*, 15166.

Loh, K.M., Ang, L.T., Zhang, J., Kumar, V., Ang, J., Auyeong, J.Q., Lee, K.L., Choo, S.H., Lim, C.Y., Nichane, M., et al. (2014). Efficient endoderm induction from human pluripotent stem cells by logically directing signals controlling lineage bifurcations. Cell Stem Cell *14*, 237–252.

Mitaka, T., Sattler, C.A., Sattler, G.L., Sargent, L.M., and Pitot, H.C. (1991). Multiple cell cycles occur in rat hepatocytes cultured in the presence of nicotinamide and epidermal growth factor. Hepatology *13*, 21–30.

Mizuguchi, T., Hui, T., Palm, K., Sugiyama, N., Mitaka, T., Demetriou, A.A., and Rozga, J. (2001). Enhanced proliferation and differentiation of rat hepatocytes cultured with bone marrow stromal cells. J. Cell. Physiol. *189*, 106–119.

Nagy, P., Bisgaard, H.C., and Thorgeirsson, S.S. (1994). Expression of hepatic transcription factors during liver development and oval cell differentiation. J. Cell Biol. *126*, 223–233.

Ohsumi, Y. (2001). Molecular dissection of autophagy: two ubiquitin-like systems. Nat. Rev. Mol. Cell Biol. *2*, 211–216.

Papazyan, R., Sun, Z., Kim, Y.H., Titchenell, P.M., Hill, D.A., Lu, W., Damle, M., Wan, M., Zhang, Y., Briggs, E.R., et al. (2016). Physiological suppression of lipotoxic liver damage by complementary actions of HDAC3 and SCAP/SREBP. Cell Metab. *24*, 863–874.

Poleshko, A., Shah, P.P., Gupta, M., Babu, A., Morley, M.P., Manderfield, L.J., Ifkovits, J.L., Calderon, D., Aghajanian, H., Sierra-Pagan, J.E., et al. (2017). Genome-nuclear lamina interactions regulate cardiac stem cell lineage restriction. Cell *171*, 573–587.e14.

Richman, R.A., Claus, T.H., Pilkis, S.J., and Friedman, D.L. (1976). Hormonal stimulation of DNA synthesis in primary cultures of adult rat hepatocytes. Proc. Natl. Acad. Sci. USA. *73*, 3589–3593.

Sapkota, G.P., Cummings, L., Newell, F.S., Armstrong, C., Bain, J., Frodin, M., Grauert, M., Hoffmann, M., Schnapp, G., Steegmaier, M., et al. (2007). BI-D1870 is a specific inhibitor of the p90 RSK (ribosomal S6 kinase) isoforms in vitro and in vivo. Biochem. J. *401*, 29–38.

Shalem, O., Sanjana, N.E., Hartenian, E., Shi, X., Scott, D.A., Mikkelson, T., Heckl, D., Ebert, B.L., Root, D.E., Doench, J.G., et al. (2014). Genome-scale CRISPR-Cas9 knockout screening in human cells. Science *343*, 84–87.

Shalem, O., Sanjana, N.E., and Zhang, F. (2015). High-throughput functional genomics using CRISPR-Cas9. Nat. Rev. Genet. *16*, 299–311.

Shan, J., Schwartz, R.E., Ross, N.T., Logan, D.J., Thomas, D., Duncan, S.A., North, T.E., Goessling, W., Carpenter, A.E., and Bhatia, S.N. (2013). Identification of small molecules for human hepatocyte expansion and iPS differentiation. Nat. Chem. Biol. *9*, 514–520.

Si-Tayeb, K., Noto, F.K., Nagaoka, M., Li, J., Battle, M.A., Duris, C., North, P.E., Dalton, S., and Duncan, S.A. (2010). Highly efficient generation of human hepatocyte-like cells from induced pluripotent stem cells. Hepatology *51*, 297–305.

Song, Z., Cai, J., Liu, Y., Zhao, D., Yong, J., Duo, S., Song, X., Guo, Y., Zhao, Y., Qin, H., et al. (2009). Efficient generation of hepatocyte-like cells from human induced pluripotent stem cells. Cell Res. *19*, 1233–1242.

Sullivan, G.J., Hay, D.C., Park, I.H., Fletcher, J., Hannoun, Z., Payne, C.M., Dalgetty, D., Black, J.R., Ross, J.A., Samuel, K., et al. (2010). Generation of functional human hepatic endoderm from human induced pluripotent stem cells. Hepatology *51*, 329–335.

Sun, Z., Miller, R.A., Patel, R.T., Chen, J., Dhir, R., Wang, H., Zhang, D., Graham, M.J., Unterman, T.G., Shulman, G.I., et al. (2012). Hepatic Hdac3 promotes gluconeogenesis by repressing lipid synthesis and sequestration. Nat. Med. *18*, 934–942.

Undevia, S.D., Kindler, H.L., Janisch, L., Olson, S.C., Schilsky, R.L., Vogelzang, N.J., Kimmel, K.A., Macek, T.A., and Ratain, M.J. (2004). A phase I study of the oral combination of CI-994, a putative histone deacetylase inhibitor, and capecitabine. Ann. Oncol. *15*, 1705–1711.

Stem Cell Reports, Volume *11*

Supplemental Information

Genetic and Chemical Screenings Identify HDAC3 as a Key Regulator in Hepatic Differentiation of Human Pluripotent Stem Cells

Shuang Li, Mushan Li, Xiaojian Liu, Yuanyuan Yang, Yuda Wei, Yanhao Chen, Yan Qiu, Tingting Zhou, Zhuanghui Feng, Danjun Ma, Jing Fang, Hao Ying, Hui Wang, Kiran Musunuru, Zhen Shao, Yongxu Zhao, and Qiurong Ding

Supplemental Information

Inventory of Supplemental Information

Supplemental Figures S1-S3 and Legends

Figure S1 related to main Figure 2

Figure S2-3 related to main Figure 3

Supplemental Experimental Procedures

Supplemental References

Supplemental Figures

Figure S1. Related to main Figure 2.

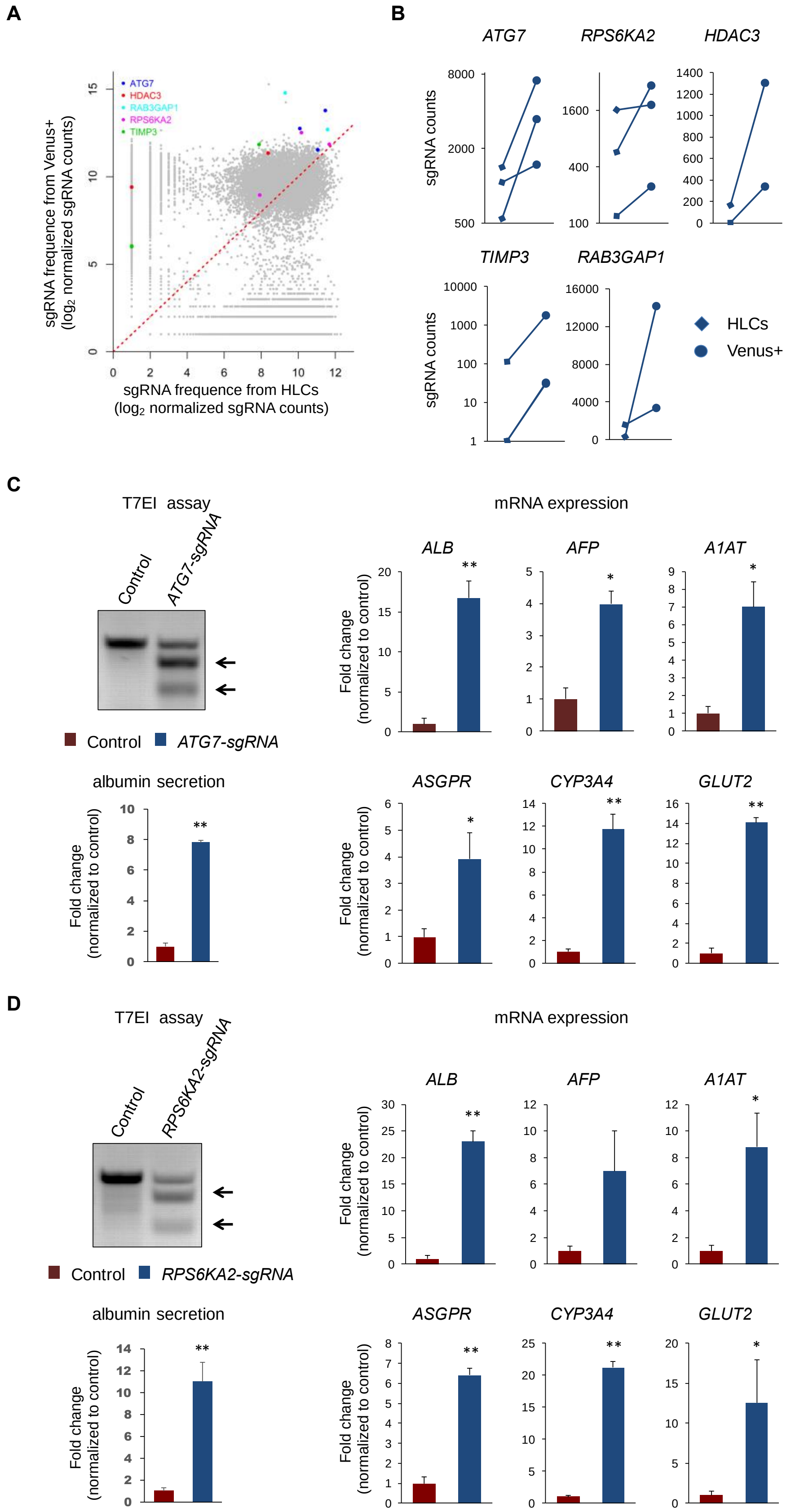

Figure S2. Related to main Figure 3.

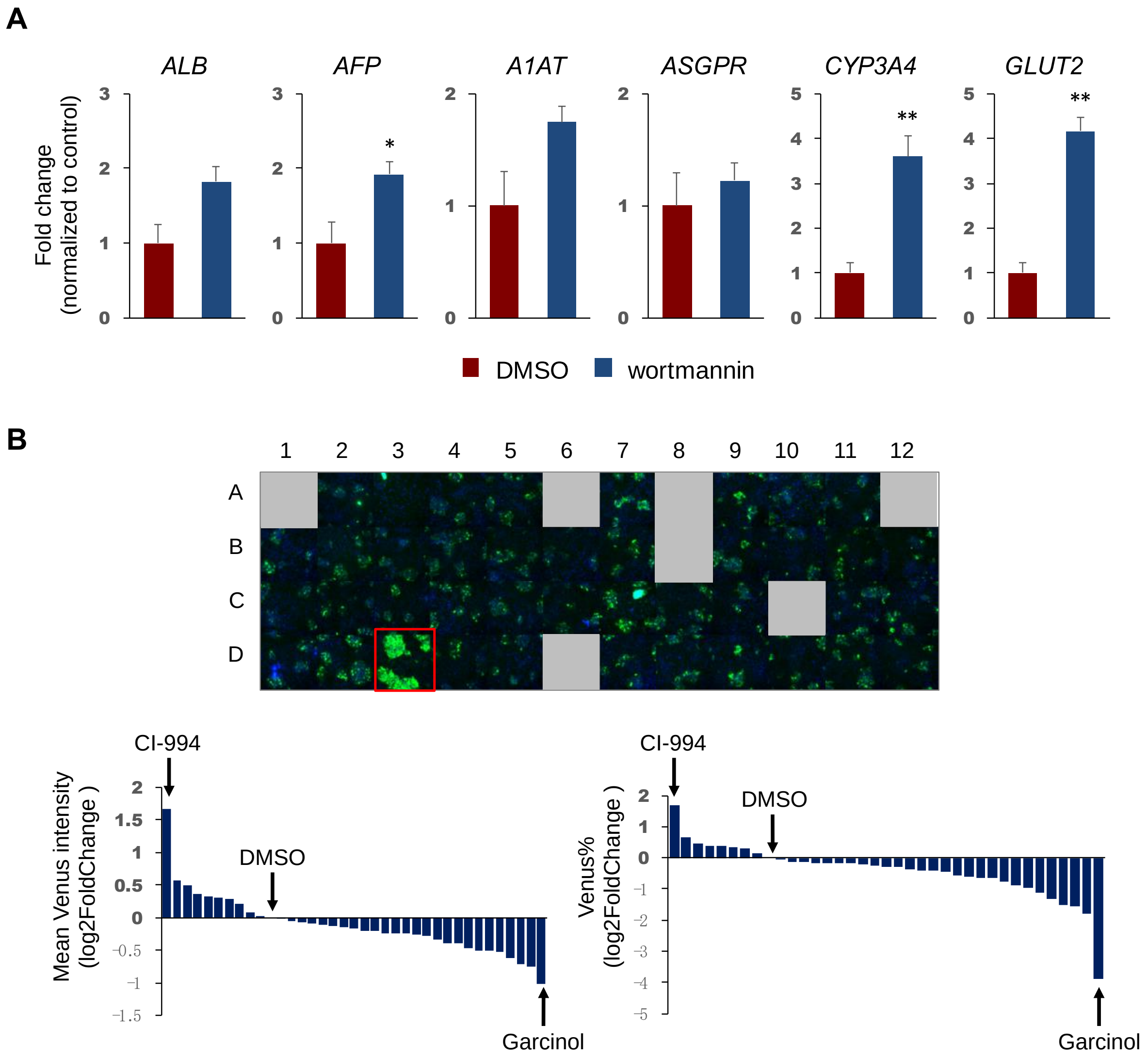

Figure S3. Related to main Figure 3.

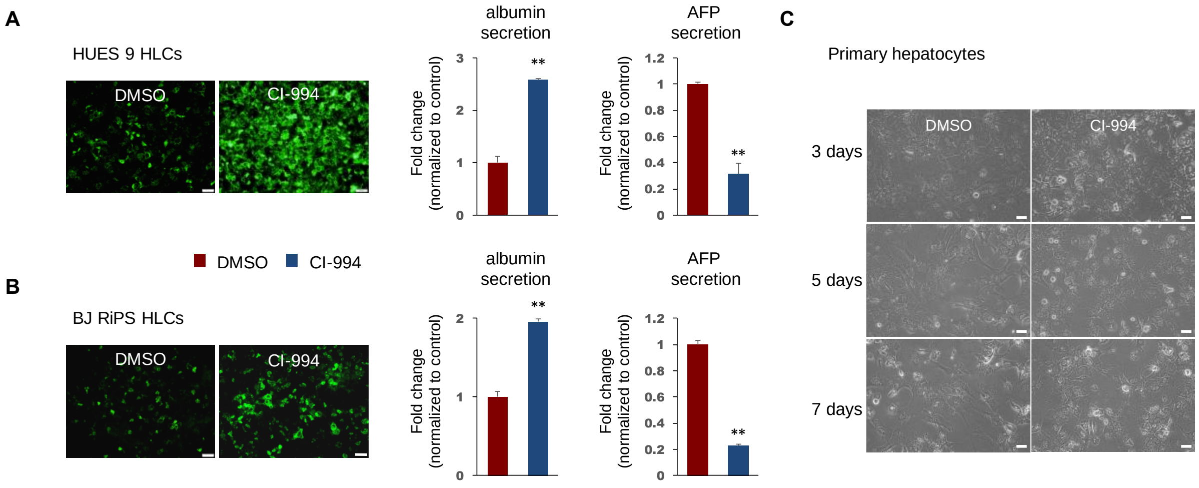

Supplemental Figure Legends

Figure S1. Genetic screenings identify several genes that are involved in regulation of HLC differentiation. Related to main Figure 2. (**A**) Scatterplot showing enrichment of specific sgRNAs in Venus+ group compared to HLC group. (**B**) sgRNA counts of indicated genes in HLC and Venus+ groups from the GeCKO screening. Counts of the third sgRNA each of *HDAC3* and *RAB3GAP1* was zero so was omitted in the graph. Two of the three sgRNAs of *TIMP3* showed similar counts and was overlapped in the graph. (**C**) T7EI analysis of HLCs treated with CRISPR-*ATG7* or control viruses. Arrows show the cleavage products resulting from the T7EI assays (above left); albumin mass measured by ELISA in media collected from HLCs treated with CRISPR-*ATG7* and control viruses, normalized to mean levels of control group (n = 3 independent experiments) (below left); gene expression analysis of CRISPR-Cas9-treated HLCs and control cells (n = 3 independent experiments) (right). (**D**) Same set of experiments as listed in (**C**) performed with CRISPR-*RPS6KA2* (n = 3 independent experiments). Data are represented as means with SEM. * P<0.05, ** P<0.01.

Figure S2. Chemical screenings identify that CI-994 improves HLC differentiation. Related to main Figure 3. (**A**) Gene expression analysis of 50 nM wortmannin-treated HLCs and control cells (n = 3 independent experiments). (**B**) Representative image of a half 96-well plate with Venus signal (green) and nuclei staining (blue) (above); mean Venus intensity (left) and percentage of Venus positive cells (right) as normalized to values in control wells treated with DMSO (below). Cells in D8-D12 wells were treated with DMSO as controls. There was no signal from some wells such as A1, A6, *etc*. due to cell death after chemical treatment. The well with CI-994 treatment was marked with red box.

Figure S3. Effects of CI-994 treatment in HLC differentiation and maintenance of primary hepatocytes in culture. Related to main Figure 3. (**A**) Representative albumin staining of HLCs derived from HUES 9 treated with 5 μM CI-994 or DMSO control (left); albumin and AFP mass measured by ELISA in media collected from HLCs treated with 10 μM CI-994 or DMSO control, normalized to mean levels of control group (right). N = 3 independent experiments. (**B**) Representative albumin staining of HLCs derived from BJ RiPS treated with 10 μM CI-994 or DMSO control (left); Albumin and AFP mass measured by ELISA in media collected from HLCs treated with 10 μM CI-994 or DMSO control, normalized to mean levels of control group (right). N = 3 independent experiments. (**C**) Representative images of primary hepatocytes in bright field treated with 10 μM CI-994 or DMSO control for indicated days (scale bar = 50 μm). Data are represented as means with SEM. * P<0.05, ** P<0.01.

Supplemental Experimental Procedures

Cell culture

Human embryonic kidney 293T cells were maintained in DMEM supplemented with 10% fetal bovine serum and 1% penicillin / streptomycin. 1016 iPSC, BJ RiPS, and HUES 9 (HSCI iPS Core, Harvard)(Cowan et al., 2004) were grown in feeder-free adherent culture in chemically defined mTeSR1 (STEMCELL Technologies, 05850) supplemented with penicillin and streptomycin. Plates were precoated with Geltrex matrix (Invitrogen, A1413202). The cells were disassociated with accutase (Invitrogen, A1110501) and regularly passaged. Mouse primary hepatocytes were isolated from 8 to 10 weeks old mice following a procedure as decribed(Dentin et al., 2004). Cells were then seed on plates precoated with collagen (Sigma, C3867-1VL) at a density of 2×10^5 per well in 12-well plates, and maintained in hepatocyte medium (Gibco, 17705-021) supplemented with 2 mM L-Glutamine (Gibco, 25030) and 2% penicillin / streptomycin. Primary human hepatocytes were purchased from BioreclamationIVT (BioIVT, M00995-P).

Lentivirus packaging

To prepare lentiviruses or the CRISPR lentivirus library, HEK293T cells in each 15-cm dish were transfected with 22.5 μg CRISPR plasmids together with 14.7 μg pMDL, 5.7 μg pRev and 7.9 μg pVSVG packaging plasmids. After transfection, medium with viral particles were collected 48 hours and 72 hours later and centrifuged at 20,000 r.p.m at 4 ℃ for 2 hours to pellet viral particles. Viral pellets were then re-suspended in DMEM at 4 ℃ overnight and titer was calculated using a PCR based titration kit (Applied Biological Materials Inc, LV900).

Construction of the *ALB-Venus* reporter line

For construction of the targeting plasmid, 500-bp homology arm directly upstream of the stop codon of human *ALB* gene was synthesized and ligated to the *Venus* cDNA sequence through a T2A sequence, which was used as 5' arm; 500-bp homology arm downstream of the stop codon of human *ALB* gene was synthesized and used as 3' arm. Both arms were subcloned into the PB-MV1Puro-TK vector (Transposagen) by Gibson assembly (NEB, E2611S). sgRNA targeting the human *ALB* gene stop codon locus (5'-AATGTGATGTTATAAGCCTA-3') was synthesized and cloned into lentiCRISPR v2 vector purchased from Addgene (#52961).

Generation of *ALB-Venus-puromycin* recombinant clones was following a similar protocol as described(Ding et al., 2013). Briefly, wild-type 1016 iPSCs were maintained as described above. For targeting, the cells were disassociated into single cells with accutase, and 10 million cells were electroporated with a mix of 30 μg of the CRISPR plasmid and 30 μg of the donor plasmid in a single cuvette (Bio-Rad). The cells were then plated and treated with 1 μg/ml puromycin for 2 days. When single colonies appeared, colonies were manually picked and replated individually to wells of 96-well plates. Colonies were allowed to grow to near confluence over the next 7 days, at which point they were split and replica-plated. Genomic DNA was extracted in 96-well format from one of the plates in lysis buffer (10 mM Tris pH 7.5, 10 mM EDTA, 10 mM NaCl, 0.5% Sarcosyl) containing proteinase K at 56 ℃ overnight in a humidified chamber. Genomic DNA was precipitated by the addition of 95% ethanol containing 75 mM NaCl for 1 hr at room temperature. The DNA was then washed two times in 70% ethanol, allowed to dry at room temperature, and then resuspended in nuclease-free water.

Genotyping to confirm the successful recombination was performed by a PCR method using 2 × Taq mixture (Transgen Biotech, AS111-11). Primer pairs used were as following: for 5' arm inserting: 5'-GGAGGCTTTGTACATGTGGG-3' (outside 5' arm) and 5'-GCTGAACTTGTGGCCGTTTA-3' (inside Venus); for 3' arm inserting: 5'-TCTTCTGTTGGGCTAGGCAA-3' (outside 3' arm) and 5'-CTAAATGCACAGCGACGGAT-3' (inside puromycin). PCR products from positive colonies were further validated by Sanger sequencing. Colonies with successful recombination were recovered from 96-well plates and expanded for puromycin cassette excision in the next step.

Excision-only piggyBac transposase expression was introduced next in positive colonies from recombination step for puromycin cassette excision. Briefly, the cells were disassociated into single cells and 10 million cells were electroporated with 30 μg pPBx-GFP plasmid (Transposagen). Seventy-two hours after electroporation, cells with GFP expression were collected by FACS and replated on 10 cm tissue-culture dishes at around 20,000 cells / dish to allow for recovery in growth media. Single colonies were then manually picked and replated individually to wells of 96-well plates. Genomic DNA and genotype of single colonies were performed as described above. Primers to confirm the successful excision of puromycin cassette were as following: 5'-AAGCTGACCCTGAAGCTCAT-3' (inside Venus) and 5'-TCTTCTGTTGGGCTAGGCAA-3' (outside 3' arm);

5'-ACCGAGCTGCAAGAACTCT-3' and 5'-TCGTAGAAGGGGAGGTTGC-3' (both inside puromycin). Colonies with successful puromycin excision were expanded for further experiments.

Genome-wide CRISPR-Cas9 screenings

The lentiviral sgRNA plasmid library for genome-wide CRISPR-Cas9 screening was purchased from Addgene (#1000000048). Library was amplified following the protocol provided by Addgene, and lentiviruses were prepared and titer was calculated as described above.

A total of 4.5×10^7 *ALB-Venus* reporter hPSCs were infected by the lentivirus library at an MOI of 0.7. Cells were treated with puromycin (1 μg/ml) for 2 days to eliminate non-infected cells. Cells were recovered for one more day after puromycin treatment, and subjected to HLC differentiation. Cells were harvested at day 5 at maturation stage and high Venus positive cells (top 5%) were collected by FASC sorting (FACSAria II; BD Biosciences). Control cells, including Venus negative cells (bottom 5%, collected by FACS sorting), HLCs (collected before FACS sorting) and infected hPSCs (collected after puromycin treatment) were obtained at indicated time points.

Genomic DNA of cells from different groups was extracted and the sgRNAs were amplified by PCR method using KOD DNA polymerase (TOYOBO, KOD-401). Briefly, in total 2.2 μg (200 ng per PCR reaction; 11 separate reactions for each sample) of genomic DNA from each group were used as DNA template; the PCR program used was 94 ℃ 5min, 98 ℃ 20s, 58 ℃ 30s, 68 ℃ 12s, 32 cycles. Products (158bp) were gel-purified and quantified. In total 1.4 μg PCR products from each group were pooled together and sent for deep sequencing (Illumina HiSeq4000 system) by using the pair-ended 150bp sequencing protocol. PCR primers used for amplification were: 5'-TGAAAGTATTTCGATTTCTTGGCTT-3', 5'-CGGTGCCACTTTTTCAAGTT-3'. An 8bp barcode for multiplexing of different biological samples were added at 5' of each primer.

For data analysis, the sequencing reads of sgRNAs from different samples were first identified by barcode using cutadapt (v1.9) with default parameters. Build-index function of Bowtie(Langmead et al., 2009) was applied on the sgRNA sequences of GeCKO library to generate Burrows-Wheeler index. The sgRNA sequences were then retrieved and counted by aligning processed reads of each sample to the sgRNA library using Bowtie. Maximum 2 mismatches were allowed and only the reads with unique alignment were reported. SgRNAs and corresponding genes significantly over- or under-represented in the samples of interest were identified by MAGeCK(Li et al., 2014) package with the median normalization option compared to the control samples.

Chemical screenings

The *ALB-Venus* reporter hPSCs were differentiated to get immature hepatocytes. Cells were then split with accutase and plated at a density around 5×10^4 cells per well in 96-well plates. After attachment, cells were cultured in maturation medium supplemented with different chemicals for 7 - 9 days. Medium was changed each day. Cells were then analyzed for Venus expression in a high-throughput platform using High Content Screening (HCS) (Cellomics ArrayScan VTI; Thermo Fisher Scientific). Chemical library was purchased from the National Compound Resource Center (Shanghai) and applied in a final concentration of 10 μM of each chemical in screening; Cells treated with DMSO were used as controls.

In experiments testing individual chemicals, immature hepatocytes differentiated from 1016 iPSC, HUES 9 or BJ RiPS as indicated were split and plated at a density around 4×10^5 cells per well in 12-well plates. Cells were then cultured in maturation medium supplemented with individual chemicals or DMSO as controls. Cells were analyzed after 7 - 9 days. Chemicals used in our study were as following: wortmannin (50 nM, Selleck, S2758), chloroquine (20 μM, Selleck, S4157), RPS6KA2 inhibitor (10 μM, Selleck, S2843), CI-994 (5 μM or 10 μM as indicated, Selleck, S2818), and RGFP966(10 μM, Selleck, S7229)

Generation of CRISPR-Cas9 knockout cell lines

The 20bp sequence of sgRNA targeting individual genes was inserted to lentiCRISPR v2 plasmid and used for lentivirus packaging. The target sequences used are 5'-TAGGGTCCATACATTCACTG-3' for human *ATG7*, 5'-GCAGGAAGAAGGCGTCGTGA-3' for human *RPS6KA2*, 5'-CAGACCACCAGCCCAGTTAA-3' for human *HDAC3-sgRNA1* and 5'-GTTGAAGGCATTAAGACTCT-3' for human *HDAC3-sgRNA2*.

Lentiviruses carrying CRISPR-Cas9 targeting individual genes or empty lentiCRISPR v2 vector as control viruses were packaged. The *ALB-Venus* reporter hPSCs were infected and selected with puromycin (1 μg/ml). Cells were next subjected to genomic DNA extraction for T7EI analysis (NEB, E3321) or to protein extraction for western blot to determine gene editing efficiency. Primers used in T7EI analysis for each gene were as following: 5'-AGGTCGTTGCTTGATCTGCT-3' and 5'-GTACAGGTACGCTGGTGGTC-3' for human *ATG7*, 5'-

GGCTCCAACGGCATTTTGTT-3' and 5'-CTCCACTTTCAAACTCCAGCG-3' for human *RPS6KA2*, 5'-AATTCCCCTCCCAGCTGC-3' and 5'-AGGGTTCCAACTTGTTCCTCT-3' for human *HDAC3-sgRNA1*, 5'-TTCCCACTGCTGCCAAAAGA-3' and 5'-TTGTGGGATGAGGGGAATGC-3' for human *HDAC3-sgRNA2*. Cells showed clear gene editing efficiency in desired targeting locus in each gene were expanded and subjected to HLC differentiation and functional analysis.

Differentiation of hPSCs into HLCs

Differentiation of hPSCs into HLCs was performed following the protocols of Si-Tayeb et al(Si-Tayeb et al., 2010). Briefly, hPSCs were 1) incubated in RPMI-B27 (RPMI-1640 from Invitrogen, 11875093; B27 supplement from Invitrogen, 12587010) medium supplemented with recombinant activin A (100 ng/mL, PeproTech, AF-120-14E) and LY-294002 (5 μM, Selleck, S1105) for 3 or 4 days to obtain definitive endoderm; 2) RPMI-B27 supplemented with BMP4 (20 ng/mL, PeproTech, 120-05) and FGF2 (5 ng/mL, PeproTech, AF-100-18B) and 0.5% DMSO for 5 days to get hepatoblasts; 3) RPMI-B27 supplemented with HGF (20 ng/mL, PeproTech, 100-39) and 0.5% DMSO for 5 days to get immature hepatocytes; and 4) HCM Hepatocyte Culture Medium (Lonza, CC-3198) supplemented with HGF (20 ng/mL), Oncostatin M (20 ng/mL, PeproTech, 300-10), dexamethasone (100 nM, Sigma, D4902) and 0.5% DMSO for 7-9 days to get mature HLCs.

ELISAs, immunocytochemistry and western blot analysis

For ELISA experiments, supernatant from differentiated HLCs or primary hepatocytes were collected at the end of differentiation (for HLCs) or on day 7 after *in vitro* culture (for primary hepatocytes) to determine the concentration of human albumin (Abcam, ab108788), human AFP (Abcam, ab193765), mouse albumin (Abcam, ab108792), respectively. Total RNA amount was used for normalization.

For immunocytochemistry analysis, differentiated HLCs or primary hepatocytes at indicated days were fixed, permeabilized with 0.1% Triton-X-100, and counterstained for albumin (Abcam, ab207327) as indicated. Nuclei were visualized with Hoechst stain (Invitrogen, H3570) and images were taken by microscopy (OLYMPUS, IX73).

For western blot analysis of *HDAC3* KO cells, differentiated HLCs treated with *HDAC3* CRISPRs or control vectors were collected. Total proteins were extracted and subjected to regular western procedure against HDAC3 (Abcam, ab32369) and tubulin (Sigma, T6557). For western blot analysis of samples in different stages during hepatic differentiation, cells were fractionated to get cytosol and nuclear sections (Thermo Scientific, 78835) and subjected to analysis with the following antibodies: HDAC3 (Abcam, ab32369), albumin (Abclonal, A0353), H3K9ac (Abcam, ab10812), H3K27ac (Abcam, ab4729), H3 (Abcam, ab10799) and tubulin (Sigma, T6557).

Quantitative RT-PCR

Total RNA was isolated from HLCs, reverse transcription and real-time PCR were performed as described[8] using standard methods. The sequences of primers were as following: 5'-GCACACTTTCTGAGAAGGAGAG-3' and 5'-CACTTCTCTACAAAAGCTGCG-3' for human *ALB* gene; 5'-TCAGTGAGGACAAACTATTGGC-3' and 5'-GGGTTTACTGGAGTCATTTCATG-3' for human *AFP* gene; 5'-GGAACCTATGATCTGAAGAGCG-3' and 5'-TGGTCAGCACAGCCTTATG-3' for human *A1AT* gene; 5'-GAGCAGAAATTTGTCCAGCAC-3' and 5'-CCTCCAGTTCTTGAAGCCC-3' for human *ASGPR* gene; 5'-TTCACCGTGACCCAAAGTAC-3' and 5'-TGAGAGCAAACCTCATGCC-3' for human *CYP3A4* gene; 5'-TTTCAGTCAAGGACCACGTC-3' and 5'-GAGCACTCCAGCAAAGAGG-3' for human *GLUT2* gene; 5'-GTCCCCAAAGAGTTTAAAGCTG-3' and 5'-ACAGTCTTCAGTTGCTCCG-3' for mouse *Alb* gene ; 5'-GCCCCTTAAACACTTGGATTG-3' and 5'-CCCATGTCCGTACCAGTTATC-3' for mouse *Asgpr* gene ; 5'-TGTGTCCCAGTTTGAATCCTC-3' and 5'-GTTATCCCAGAAGTCCCGAG-3' for mouse *Apoa1* gene ; 5'-AAAACAGTTGGAGCAAAGGC-3' and 5'-CAAGGGTCCCAGCTTTTCTAG -3' for mouse *Apoa5* gene ; 5'-AGGAGTCCGATATAGCTGTGG-3' and 5'-CTCACGACTCAATAGCTGGAG-3' for mouse *Apoc3* gene ; 5'-TTGGCAAGGTGATGGAAGAG-3' and 5'-TCTCTGTAAGATAGGCCTCCC -3' for mouse *Arg1* gene ; 5'-CAATCCCTTGGTTCATGGTTG-3' and 5'-AGGAAGTCCGCAATGTACTG-3' for mouse *Glut2* gene ; 5'-GCCAACTTTGCTCGGAATG-3' and 5'-TCCACTTCCTTGTCTTTCAGC-3' for mouse *Ces1g* gene ; 5'-CGGCTACCACATCCAAGGAA-3' and 5'-GCTGGAATTACCGCGGCT-3' for 18S RNA.

Plasmids and Co-immunoprecipitation

The plasmids of HA-HNF4 and HDAC3-Flag were constructed by standard molecular cloning techniques. The *HNF4* gene was amplified and cloned into the pCDH vector (System Biosciences, CD527A-1) with an HA-tag inserted to 5' of the cDNA. The *HDAC3* gene was constructed into pcDNA3.0 vector with a FLAG-tag at 3' of

the cDNA. These two plasmids were co-transfected into HEK293T cells using Lipofectamine 2000 (Invitrogen, 11668019) following the manual protocol. Forty-eight hours after transfection, HEK293T cells were lysed with the RIPA buffer (Millipore, 20-188). The whole cell lysate was then immunoprecipitated with anti-M2 beads (Sigma, A2220) at 4 ℃ overnight. Beads were then washed three times with the RIPA buffer, and bound HNF4 was immunoblotted with an anti-HA antibody (Cell Signaling Technology, 3724).

Dual-Luciferase reporter assay

The human *ALB* promoter/enhancer region containing HNF4 binding sites (-7264～-5992 from *ALB* TSS) was amplified from iPSC genomic DNA and cloned into the pGL3-basic luciferase vector (Promega, E1751). Luciferase assays were carried out by transiently transfecting HEK293T cells using Lipofectamine 2000. Briefly, HEK293T cells were seeded into 24-well plates and transfected with 100 ng of indicated luciferase reporter constructs and 10 ng pRL-TK in each well together with 200 ng HNF4 and/or 200 ng HDAC3. The empty vector pCDH was selected to maintain equal amounts of DNA among wells. Cells were lysed 24 hours post-transfection and analyzed for luciferase activity with the dual-luciferase assay Kit (Promega, E1910) according to the manufacturer’s protocol.

Chromatin immunoprecipitation

ChIP assays were carried out using the SimpleChIP® Enzymatic ChIP Kit (Cell Signaling Technology, 9002) following the manufacturer’s protocol. Briefly, HLCs (2×10^7cells) treated with CI-994 or DMSO were fixed with 1% formaldehyde for 10min at room temperature and quenched with glycine for 5 min. The cells were then lysed with the lysis buffer. Chromatin was later digested at 37 ℃ for 10min to generate fragments with an average length of 150-900 bp. The samples were then sonicated on ice and supernatant was transferred to a new tube. 2% supernatant was saved for input at this stage, and the remnant was subjected to immunoprecipitation at 4 ℃ overnight with the following antibodies, respectively: anti-HNF4a (Abcam, ab181604), anti-H3K9ac (Abcam, ab10799), anti-H3K27ac (Abcam, ab4729) and IgG (Cell Signaling Technology, 2729) as control. Each reaction was then incubated with protein G agarose beads for another 2 hours at 4 ℃. After standard washes, elution buffer was added to all immunoprecipitation samples and the input samples. DNA from each sample was then purified for real-time PCR. Primers used in the real-time PCR were as following: 5’- AATGGGAATTAGTACTGGTTTGGGA-3’, and 5’-ACAGTCATTGCCCAGAATGTTAC-3’ for anti-HNF4; 5’-CTGAACAGCCAAACAGAGATTCC-3’, and 5’-ACCATGGGAATCCTATCCCACTA-3’ for anti-H3K9ac and anti-H3K27ac. Fold enrichment was then calculated as: Percent Input = $2\% \times 2^{(C[T]\ 2\%\text{Input Sample} - C[T]\ \text{IP Sample})}$; C[T] = CT = Threshold cycle of PCR reaction.

RNA-seq analysis

Total RNA from differentiated HLCs treated with CI-994 or DMSO control was extracted and subjected to quality assessment. In total six samples were prepared with three replicates in either group and sequencing was performed in WuXi Apptec Co. Ltd (Shanghai). For data analysis, the raw RNA-seq reads were aligned to human genome (hg19) by STAR. The number of reads for each RefSeq gene was counted by HTSeq(Pruitt et al., 2007). Differentially expressed genes were then identified by DEseq(Anders and Huber, 2010) by using fold change ≥ 2 and FDR ≤ 0.01 as cutoff. GO term and pathway annotations were downloaded from Gene Ontology website. GO enrichment analysis were performed by homemade scripts of R and Python scripts using Fisher's exact test.

Statistical analysis

The unpaired, two-tailed Student’s t test was used for experiments with two groups, and one-way ANOVA test and post-hoc Bonferronic multiple-comparison test was used for experiments that contained more than two groups. All data are represented as means with SEM.

Supplemental References

Anders, S., and Huber, W. (2010). Differential expression analysis for sequence count data. Genome Biol *11*, R106.

Cowan, C.A., Klimanskaya, I., McMahon, J., Atienza, J., Witmyer, J., Zucker, J.P., Wang, S., Morton, C.C., McMahon, A.P., Powers, D., et al. (2004). Derivation of embryonic stem-cell lines from human blastocysts. N Engl J Med 350, 1353-1356.

Dentin, R., Pegorier, J.P., Benhamed, F., Foufelle, F., Ferre, P., Fauveau, V., Magnuson, M.A., Girard, J., and Postic, C. (2004). Hepatic glucokinase is required for the synergistic action of ChREBP and SREBP-1c on glycolytic and lipogenic gene expression. J Biol Chem *279*, 20314-20326.

Ding, Q., Lee, Y.K., Schaefer, E.A., Peters, D.T., Veres, A., Kim, K., Kuperwasser, N., Motola, D.L., Meissner,

T.B., Hendriks, W.T., *et al.* (2013). A TALEN genome-editing system for generating human stem cell-based disease models. Cell Stem Cell *12*, 238-251.

Langmead, B., Trapnell, C., Pop, M., and Salzberg, S.L. (2009). Ultrafast and memory-efficient alignment of short DNA sequences to the human genome. Genome Biol *10*, R25.

Li, W., Xu, H., Xiao, T., Cong, L., Love, M.I., Zhang, F., Irizarry, R.A., Liu, J.S., Brown, M., and Liu, X.S. (2014). MAGeCK enables robust identification of essential genes from genome-scale CRISPR/Cas9 knockout screens. Genome Biol *15*, 554.

Pruitt, K.D., Tatusova, T., and Maglott, D.R. (2007). NCBI reference sequences (RefSeq): a curated non-redundant sequence database of genomes, transcripts and proteins. Nucleic Acids Res *35*, D61-65.

Si-Tayeb, K., Noto, F.K., Nagaoka, M., Li, J., Battle, M.A., Duris, C., North, P.E., Dalton, S., and Duncan, S.A. (2010). Highly efficient generation of human hepatocyte-like cells from induced pluripotent stem cells. Hepatology *51*, 297-305.
